# Cardiovascular disease and its management among Pacific people: a systematic review by ethnicity and place of birth

**DOI:** 10.1186/s12872-021-02313-x

**Published:** 2021-10-24

**Authors:** Julie Winter-Smith, Vanessa Selak, Matire Harwood, Shanthi Ameratunga, Corina Grey

**Affiliations:** 1 Section of Epidemiology and Biostatistics, Faculty of Medical and Health Sciences, The University of Auckland, Auckland Mail Centre, Private Bag 92019, Auckland, 1142 New Zealand; 2 Department of General Practice and Primary Health Care, Faculty of Medical and Health Sciences, The University of Auckland, Auckland Mail Centre, Private Bag 92019, Auckland, 1142 New Zealand; 3 Population Health Directorate, Counties Manukau Health, Private Bag 93311, Otahuhu, Auckland, 1640 New Zealand; 4 Performance Improvement, Auckland District Health Board, Auckland Mail Centre, Private Bag 92189, Auckland, 1142 New Zealand

**Keywords:** Cardiovascular disease, Atherosclerosis, Pacific, Migrant, Epidemiology, Management, Equity

## Abstract

**Background:**

Pacific people experience a disproportionate burden of cardiovascular disease (CVD), whether they remain in their country of origin or migrate to higher-income countries, such as Australia, Aotearoa New Zealand or the United States of America. We sought to determine whether the CVD health needs of Pacific people vary according to their ethnicity or place of birth.

**Methods:**

We conducted a systematic review of medical research databases and grey literature to identify relevant data published up to 2020. Texts were included if they contained original data stratified by Pacific-specific ethnicity or place of birth on the burden or management of CVD, and were assessed as having good quality using a National Heart, Lung, and Blood Institute quality assessment tool. The protocol for this review was registered with the Open Science Forum (https://doi.org/10.17605/OSF.IO/X7NR6).

**Results:**

Of 3679 texts identified, 310 full texts were reviewed and the quality of 23 of these assessed, using the pre-defined search strategy. Six items (four reports, one article, one webpage) of good quality met the review eligibility criteria. All included texts provided data on epidemiology but only one reported on the management of CVD. Four texts were of Pacific populations in Pacific Island countries and two were of Pacific diaspora in other countries. Data from the Global Burden of Disease study, which provided estimates for the greatest number of Pacific countries, showed substantial differences in mortality rates between Pacific countries for every CVD type. For example, the mortality rate per 100,000 for ischemic heart disease (IHD) ranged from 103.41 in the Cook Islands to 430.35 in the Solomon Islands. A New Zealand-based report showed differences in CVD rates by Pacific ethnicity (e.g. the age-standardised prevalence of IHD per 1,000 population in Auckland ranged from 107.8 (Niuean) to 138 among Cook Islands Māori (p < 0.001)).

**Conclusions:**

This review of published studies reveals that the epidemiology of CVD among Pacific people varies by specific ethnic groups, place of birth, and country of residence. There is a critical need for high-quality contemporary ethnic-specific Pacific data to respond to the diverse CVD health needs in these underrepresented groups.

**Supplementary Information:**

The online version contains supplementary material available at 10.1186/s12872-021-02313-x.

## Background

There is a high burden of cardiovascular disease (CVD) among Pacific people globally [1–3]. CVD, which is mainly comprised of ischaemic heart disease (IHD) (e.g. myocardial infarction), cerebrovascular disease (CeVD) (e.g. stroke) and peripheral vascular disease (PVD) (e.g. intermittent claudication), is the major cause of the difference in life expectancy between Pacific and non-Pacific people in New Zealand. Along with diabetes, CVD is the major cause of amenable mortality among Pacific peoples [1, 4]. In NZ, the amenable mortality rate from CVD among Pacific men is 2.5 times the rate of all NZ men, and for Pacific women it is 3.2 times the rate for NZ European women [4]. Despite overall reductions in the morbidity and mortality of CVD in New Zealand since the 1980s among all ethnic groups in NZ, Pacific people continue to experience a disproportionately high burden of CVD compared with Europeans [5].

There is an increasing recognition of the importance of understanding and addressing the health needs of Pacific people in NZ, Australia and the USA [6–8]. However, health systems in these nations generally consider the needs of Pacific people as a homogenous Pacific group, despite being made up of heterogeneous Pacific groups [1], both in terms of ethnicity and country of birth. Such generalisation has the potential to mask Pacific-specific health needs and inequities between different Pacific groups, and undermine the appropriateness of homogeneous, “one size fits all”, Pacific health services [1].

A 2012 health needs analysis of Pacific peoples in Auckland, in which 71% of the New Zealand Pacific population resides, assessed a wide range of the health outcomes of the five largest Pacific subgroups [2]. That analysis found that the prevalence of IHD and PVD were significantly higher among Cook Islands Māori than among other Pacific subgroups [2]. Conversely, the prevalence of stroke was higher in Samoans and lower in Tongans, compared with other Pacific peoples [2]. There is also some evidence that the all cause- and cancer- and CVD-related mortality rates for Pacific migrants resident in NZ for fewer than 25 years are lower than those resident for more than 25 years [9].

In the absence of a comprehensive investigation of the topic, this systematic review sought to determine whether there are differences in the epidemiology and management of CVD among Pacific people according to Pacific-specific ethnicity or place of birth. As a group of authors resident in NZ, we were particularly interested in the evidence relating to Pacific ethnic groups in NZ as well as in Pacific people in other countries.

## Methods

### Study design and eligibility criteria

We undertook a systematic review, reported here using the Preferred Reporting Items for Systematic Reviews and Meta-Analyses (PRISMA) guideline [10]. The review was prospectively registered on Open Science Forum in March 2020 (https://doi.org/10.17605/OSF.IO/X7NR6).

We included published original studies and systematic reviews, and reports of governments / global public health organisations that presented observational data (cross sectional, cohort, case–control) on Pacific subgroup CVD epidemiology or management. We excluded unpublished theses. There were no restrictions on study setting, publication date, or language.

Studies examining atherosclerotic CVD, atrial fibrillation (AF), hypertensive heart disease, or heart failure epidemiology or management for people of at least two Pacific-specific ethnic groups (for differences by Pacific-specific ethnicity) or one Pacific-specific ethnic group (for differences by place (country or region) of birth) were included. We excluded studies of CVD other than atherosclerotic CVD, AF, hypertensive heart disease or heart failure (e.g. rheumatic heart disease, congenital heart disease, cardiomyopathy), and excluded studies which focussed on a broader Pacific group (e.g. Pacific Islanders).

Included Pacific-specific ethnic groups were defined as ethnic groups that are indigenous to the South Pacific sub regions of Niue, Cook Islands, Fiji, Hawaii, Tokelau, Kiribati, Tuvalu, Tahiti, Wallis and Futuna, Nauru, PNG, Solomon Islands, Vanuatu, French Polynesia, Tonga, New Caledonia, Guam, Federated States of Micronesia, Palau, Pitcairn Island, Samoa, Marshall Islands, Easter Island, Northern Mariana Islands, Rotuma (as specified by the Pacific Community (formerly South Pacific Commission) and guidance from a Pacific librarian) [11].

The outcomes sought were differences in the epidemiology and management of CVD among Pacific people according to Pacific-specific ethnicity and place of birth. The epidemiology outcomes were CVD mortality (primary outcome), hospitalisations, and incidence or prevalence (composite, by type). The management outcomes were gaps in the use of the following interventions among people with established CVD: triple therapy (antiplatelet, blood pressure lowering and lipid lowering therapy) (primary outcome), triple therapy components and cardiovascular investigations and interventions (specifically angiography, percutaneous coronary intervention [PCI, with or without stenting] and coronary artery bypass grafts [CABG]). Medication “use” included prescription and dispensing as well as adherence to medication.

### Information sources and search strategy

We conducted a search on MEDLINE (OVID), EMBASE, Scopus, PubMed and the Cochrane Database of Systematic Reviews up to 24 March 2020. Terms (and synonyms of) ‘atherosclerosis’ or any of the atherosclerotic cardiovascular diseases (ischemic heart diseases, cerebrovascular diseases, peripheral vascular diseases) or cardiovascular diseases related to atherosclerosis (heart failure, hypertensive heart disease, atrial fibrillation), ‘epidemiology’ and ‘treatment, adherence and compliance’ were combined with ‘Pacific people’, any of the Pacific regions or individual Pacific countries. MESH terms were used for each of ‘atherosclerosis’, atherosclerotic cardiovascular diseases, ‘epidemiology’, ‘treatment, adherence and compliance’, ‘Pacific people’, Pacific regions, individual Pacific countries. Full details of our search strategy can be found in Additional file 1: Figs. S1–S5.

Two researchers (JW, VS) also searched for reports of governments and global public health organisations that presented observational data (cross sectional, cohort, case–control) on Pacific subgroup CVD epidemiology or management independently to identify those meeting our inclusion criteria. Title and contents pages were initially searched, before screening the relevant chapters. Disagreements about whether the grey literature met the inclusion criteria were resolved through discussion about the reasoning for their decisions. Full details on government and global public health organisation webpages that were searched are noted in Additional file 1: Fig. S6.

Reference lists of each of the full texts originally assessed for eligibility were also checked for potential citations to include in this systematic review.

### Data extraction and quality assessment

References were imported into Covidence (www.covidence.org, Veritas Health Innovation Ltd.), which was used to manage the initial title and abstract screening. EndNote was then used to manage full-text screening. Two researchers (JW, VS) screened all titles, abstracts and full texts independently to identify those meeting our inclusion criteria. For disagreements about whether the texts met the inclusion criteria, JW and VS reviewed these titles, abstracts and full texts together, discussed reasoning for their decisions and resolved disagreements. Two other researchers (MH, CG) were available to adjudicate any unresolved disagreements, but this was not needed during the selection process. Neither of the review authors were blind to the journal titles, study authors, or institutions.

For quality assessment, we used the National Heart, Lung, and Blood Institute (NHLBI) Quality Assessment Tool for Observational Cohort and Cross-sectional studies. The authors chose to use the NHLBI tool as we judged it to be able to be applied to both peer-reviewed academic literature and grey literature reporting observational data. Each study was independently assessed by two reviewers (JW, VS) using the NHLBI standardised quality assessment tool. Studies were ranked as ‘yes’ or ‘no’ with regard to selection bias, performance bias, reporting bias and then given an overall judgement of ‘poor’, ‘fair’ or ‘good’. Good quality references were defined as having methodological rigour to enable the evaluation of the internal validity of the broad type of included studies and reports. Where there was disagreement, JW and VS discussed and came to an agreement of the rankings and overall judgement. Two researchers (JW and VS) independently extracted data and discrepancies were resolved by a third reviewer.

## Results

**Fig. 1:**
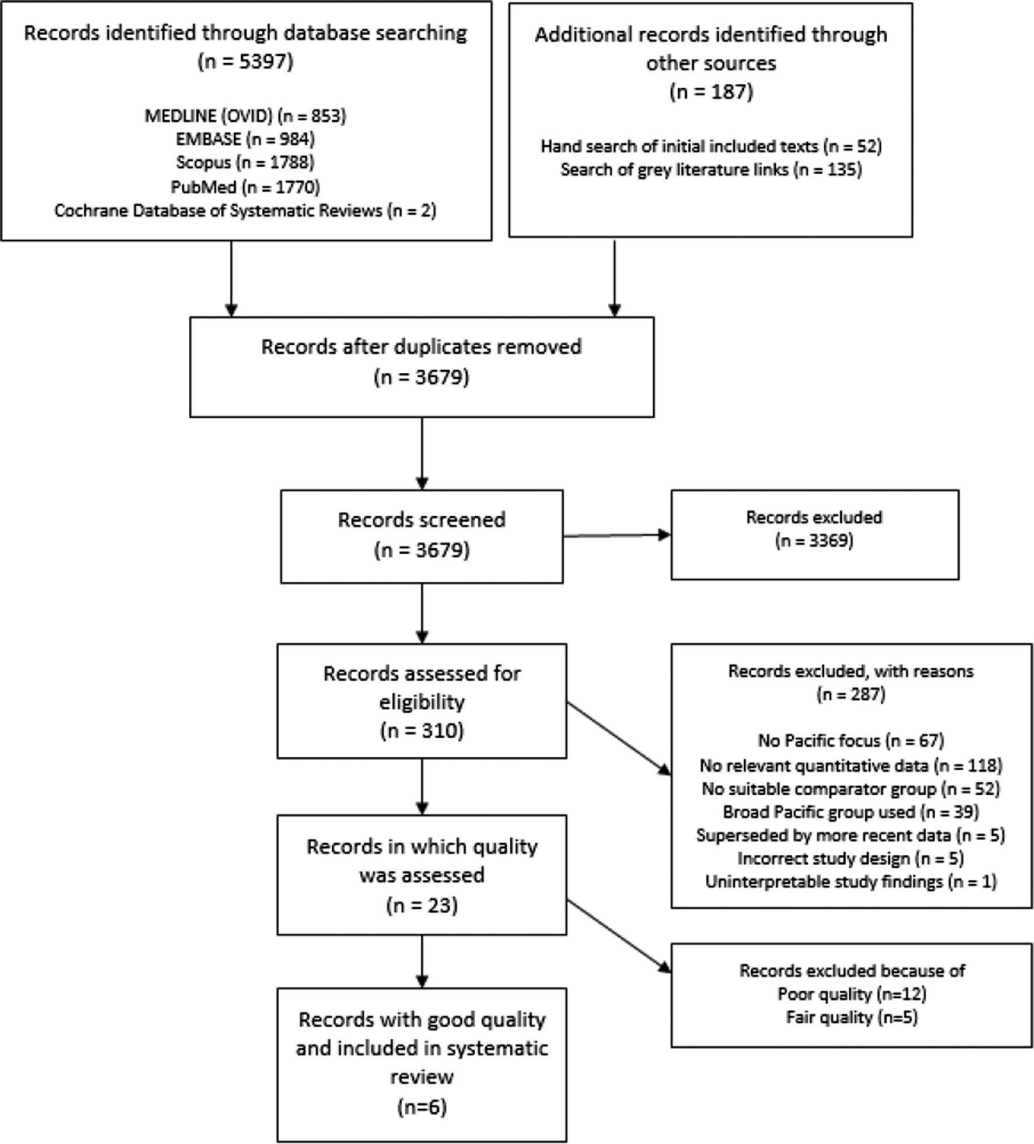
PRISMA diagram

Figure 1, below, gives details of the number of records identified, screened, assessed for eligibility and included in this systematic review. The 23 texts reviewed for quality were all cross sectional and included a series of papers by the same research group, in which the same methods were used but outcomes reported separately for different countries [12–20].

Most of the studies in which quality was assessed were of poor (n = 12) or fair (n = 5) quality, with only six texts assessed to be of good quality [1–3, 21–23]. The full quality assessment of each study using the NHLBI tool (along with summary information for each study) is provided in Additional file 2: Tables S1 and S2. The major quality issues in the fair and poor quality texts were selection bias, information bias, and confounding. There was missing information on how participants were recruited, or differences in participant recruitment across sites. Comparisons were made between groups that were not uniform/comparable and numerator-denominator bias was present. In some instances, CVD outcomes measured were presented as counts or crude data only.

### Included studies

Of the six included texts (four reports, one webpage, and one article), four were of Pacific populations in Pacific Island countries and two were of Pacific diaspora in other countries (Table 1) [1, 2]. All included studies reported data on CVD epidemiology [1–3, 21–23], but only 1 reported data on CVD management [23].

**Table 1:** Characteristics of included studies and reports

First author, year	Location(s)	Data Source/Study Population, year(s)	Population characteristics: age, sex, median annual income	Country (international) or ethnicity (New Zealand)	Cardiovascular outcomes reported and included in systematic review
International					
GBD 2019 [3]	Countries listed under Oceania	Global Burden of Disease Study 2019 data resource	All ages, both sexes, no income data reported	American Samoa, Guam, Marshall Islands, Cook Islands, Federated States of Micronesia, Kiribati, Northern Mariana Islands, Papua New Guinea, Nauru, Vanuatu, Niue, Tonga, Palau, Tokelau, Tuvalu, Solomon Islands, Samoa	Epidemiology • Incidence: atrial fibrillation and flutter, hypertensive heart disease, intracerebral haemorrhage, ischemic heart disease, ischemic stroke, peripheral vascular disease, stroke, subarachnoid haemorrhage • Prevalence: as for incidence plus hypertensive heart disease • Mortality: as for prevalence
Mendis 2011 [21]	Global	Global burden of disease estimates	All ages, both sexes, no income data reported	Cook Islands, Kiribati, Marshall Islands, Federated States of Micronesia, Nauru, Niue, Palau, Papua New Guinea, Samoa, Solomon Islands, Tonga, Tuvalu, Vanuatu	Epidemiology • Mortality: stroke, ischemic heart disease
WHO 2014 [22]	Global	-	Under age 70, males and females, no income data reported	Papua New Guinea, Solomon Islands	Epidemiology • Mortality: composite
WHO 2018 [23]	Global	Life estimates derived from routinely collected data 2000–2016	Age not specified, males and females, no income data reported	Kiribati, Nauru, Solomon Islands, Tuvalu	Epidemiology • Incidence: composite • Management: heart attacks and stroke combined
New Zealand					
Blakely 2009 [1]	New Zealand	New Zealand Census-Mortality Study 2001–2004	0–74 years old, sexes combined, income ranging from NZD $17,500 to $21,500	Samoan, Cook Island, Niuean, Tongan	Epidemiology • Mortality: composite
Health Partners Consulting Group 2012 [2]	Metro-Auckland (Counties Manukau, Auckland, Waitemata), New Zealand	National surveys, the PRIMHD database, clinical network data from the Northern Region DHBs	15 years and over, men and women, no income data reported	Samoan, Cook Island Maori, Tongan, Niuean	Epidemiology • Prevalence: ischemic heart disease, stroke, heart failure, peripheral vascular disease

The results are reported according to comparisons by Pacific country using the international studies, and by Pacific ethnicity using the NZ studies.

### International studies (comparisons by Pacific country)

#### CVD mortality

Only one study (WHO 2014) provided a comparison of composite CVD mortality rates among different Pacific countries [22]. In this study the age-standardised mortality rate for CVD (per 100,000 people) was higher for people from the Solomon Islands (men 314.6, women 196.9) than those from PNG (men 179.4, women 125.3) [22]. No confidence intervals were provided with these estimates.

Age-standardized mortality rate was reported, by CVD type, per 100,000 population in 2019, for 17 Pacific countries by the Global Burden of Disease (GBD) study [3]. Rates of CVD were lowest in Guam. The IHD mortality rate was more than four times higher in Solomon Islands (430.35.

(95% CI 358.68, 501.14)) than in the Cook Islands (103.41 (87.32, 122.36)) [3]. Hypertensive Heart Disease mortality was twenty times higher in the Cook Islands (65.27 (54.30, 78.06)) than in Palau (3.16 (2.40, 3.97)) [3]. Stroke mortality was more than six times higher in the Solomon Islands (308.08 (259.94, 360.42)) than in Guam (50.60 (43.00, 59.92)) [3]. Mortality due to intracerebral haemorrhage was more than eight times higher in the Solomon Islands (430.35 (358.68, 501.14)) than in Guam (25.32 (21.24, 30.10)) [3]. Ischemic stroke mortality was more than three times higher in Nauru (75.90 (60.51, 93.19)) than in Guam (21.51 (17.99, 25.50)) [3]. Mortality due to subarachnoid haemorrhage was more than six times higher in the Solomon Islands (19.54 (11.53, 33.01)) than in the Cook Islands (3.15 (2.08, 4.63)) [3]. Mortality due to PVD was more than ten times higher in the Northern Mariana Islands (0.60 (0.40, 0.77)) than in Palau (0.09 (0.06, 0.13)) [3]. Mortality due to AF and flutter was more than two times higher in the Federated States of Micronesia (7.08 (5.45, 9.35)) than in Guam (3.10 (2.52, 3.72)) [3].

Age-standardized mortality of two types of CVD, stroke and IHD, were reported by the WHO for 13 Pacific countries in 2008 (Mendis 2011) [21]. IHD mortality was more than 20 times higher in the Marshall Islands (237.7) than in Kiribati (11.8), with the Solomon Islands (97.9) and Cook Islands (79.1), the countries reported as the highest and lowest mortality attributed to IHD in the GBD 2019 study, having similar mortality and rates closer to that of Kiribati [21]. Stroke mortality was over three times higher in the Marshall Islands (240.4) than in the Cook Islands (71.1), and over two times higher than in the Solomon Islands (105.5) [21]. No confidence interval was provided to indicate the degree of uncertainty in these estimates [21]. Full details of mortality data extracted from all records assessed for quality can be found in Additional file 2: Table S3.

#### Incidence of CVD

Only one study (GBD study) provided a comparison of the incidence and prevalence of CVD (by type, by 100,000) among 17 different Pacific countries.

Age-standardized IHD incidence was highest in Vanuatu (225.45 (195.06, 256.61)) and lowest in the Federated States of Micronesia (187.54 (161.42, 215.21)) [3]. The incidence of stroke in Kiribati (296.11 (278.46, 316.57)) was almost twice as high as that in Tonga (151.01 (138.04, 165.29)) [3]. Ischemic stroke incidence was highest in Nauru (122.43 (106.73, 140.81)) and lowest in PNG (72.44 (62.38, 84.28)) [3]. The incidence of intracerebral haemorrhage in Kiribati (161.75 (150.73, 173.76)) was almost three times the rate of that in Guam (55.07 (49.68, 61.18)) [3]. The incidence of subarachnoid haemorrhage was almost two times higher in Kiribati (25.55 (22.61, 28.86)) than in Niue 15.35 (13.29, 18.48)) [3]. The incidence of PVD was highest in Kiribati (192.39 (166.58, 220.88)) and lowest in Guam (124.09 (107.36, 142.57)) [3]. The incidence of AF and atrial flutter was the most similar across all of the Pacific countries, with it being the highest in Vanuatu (64.46 (48.86, 82.64)) and lowest in the Federated States of Micronesia (57.05 (42.95, 73.12)) [3].

#### CVD / high risk of CVD prevalence

One text provided a comparison of the proportion of the population at high risk for CVD or with existing CVD combined between four different Pacific Island nations [23]. The reported proportions were highest in Nauru (25%), and lowest in the Solomon Islands (9%), with the proportions in Kiribati (11%) and Tuvalu (13%) being at the lower end [23]. The prevalence of the population at high risk for CVD or with existing CVD was roughly 1.9 to 2.8 times higher in Nauru than in Tuvalu, Kiribati and the Solomon Islands.

#### CVD prevalence

The age-standardized prevalence of IHD was highest in Vanuatu (2738.31 (2412.48, 3139.68)) and lowest in the Federated States of Micronesia (2,185.09 (1,924.90, 2,508.65)) [3]. The prevalence of hypertensive heart disease was almost nine times higher in the Cook Islands (703.08 (532.87, 920.72)) than in Palau (79.37 (57.00, 110.93)) [3]. Stroke prevalence was 1.6 times higher in Kiribati (2,263.12 (2,123.52, 2,401.71)) than in Tonga (1,416.83 (1,322.55, 1,519.59)) [3]. Ischemic stroke prevalence was higher in Nauru (1,360.73 (1,241.00, 1,500.20)) than in PNG (860.87 (782.83, 942.65)) [3]. The prevalence of intracerebral haemorrhage was over two times higher in Kiribati (971.74 (869.97, 1067.69) than in Tonga (394.32 (355.70, 437.63)) [3]. The prevalence of subarachnoid haemorrhage was highest in Kiribati (194.47 (171.62, 219.55)) and lowest in Palau (130.70 (113.77, 150.33)) [3]. The prevalence of PVD was highest in Kiribati (2,009.41(1,734.25, 2,306.45)) and lowest in PNG (1,356.00 (1,173.62, 1,550.64)) [3]. The prevalence of AF and atrial flutter ranged from 703.06 (531.31, 906.31) in the Federated States of Micronesia to 806.20 (608.34, 1040.39) in American Samoa [3].

Full details of incidence and prevalence data (composite, by type) extracted from all records assessed for quality can be found in Additional file 2: Tables S5 and S6.

#### CVD management

Only one text compared use of appropriate CVD management according to CVD risk across Pacific countries [23]. That text found that the proportion of people with established CVD receiving appropriate CVD medication or therapy was almost four times higher in Tuvalu (43%) and almost three times higher in Nauru (30%) and Kiribati (29%) when compared to that of the Solomon Islands (11%) [23].

Full details of management data extracted from all records assessed for quality can be found in Additional file 2: Tables S7 and S8.

### New Zealand studies (comparisons by Pacific ethnicity)

#### CVD mortality

Among Pacific diaspora, only one study (Blakely 2009) provided a comparison of composite CVD mortality rates among different Pacific ethnic groups [1]. That study found that the mortality rate for CVD among Pacific people in New Zealand was highest among Cook Islands Māori (111 (90, 135) per 100,000 people), followed by Niuean (75 (51,104)), Samoan (68 (57, 79)) and lowest among the Tongan population (58 (40, 78)) [1]. The differences in CVD mortality between Tongan and Cook Islands Māori ethnicities present a stark picture as the 95% CIs do not overlap and the Cook Islands Māori rate is over 91% higher than the rate for the Tongan ethnic group.

#### CVD prevalence

Only one study (HPCG 2012) provided a comparison of the prevalence of CVD by type among different Pacific ethnic groups resident in NZ [2]. Prevalence for each CVD type was reported by Pacific-specific group as well as for all Pacific per 1,000 age-standardised population (*P* < 0.001) in the metro-Auckland area in 2011. That study estimated that the prevalence of IHD for all Pacific was 118 (per 1,000 age-standardised population (*P* < 0.001)) and 92 for non-Māori non-Pacific people [2]. The prevalence of IHD was highest among Cook Islands Māori (138), followed by Samoan (118), Tongan (113), and lowest in the Niuean ethnic group (107.8) [2].

The estimated prevalence of stroke for all Pacific was 1.5 times higher (12.1 per 1,000 age-standardised population (*P* < 0.001)) than for non-Māori non-Pacific (7.9) [2]. The reported prevalence was highest in Samoan people (13.5 (*P* < 0.05)), followed by Cook Islands Māori (12.9), Niueans (10.3), and lowest in Tongans (9.9 (*P* < 0.05)) [2].

The estimated prevalence of PVD among all Pacific (5.3 per 1,000 age-standardised population) was 1.3 times higher than in the non-Māori non-Pacific population (3.9) [2]. The reported PVD prevalence was highest in Cook Islands Māori (7.0 (*P* < 0.05)), followed by Niuean (5.3), Tongan (5.1) and then Samoan ethnic groups (4.9) [2]. The rate ratio for PVD prevalence between Cook Islands Māori and non-Māori non-Pacific was almost 1.8. This is significantly more than the rate ratio for PVD prevalence between Samoan and non-Māori non-Pacific of approximately 1.25 [2].

The estimated prevalence of heart failure was more than twice as high in Pacific people (24.2 per 1,000 age-standardised population (*P* < 0.001)) compared to non-Māori non-Pacific (11.6) people [2]. Differences in the prevalence of heart failure existed between Pacific-specific groups. The prevalence of heart failure among Cook Islands Māori (34.5) was more than 1.4 times the prevalence in Tongans (23.8) and Samoans (23.3) and 1.7 times higher than in Niueans (20.3 (*P* < 0.05))[2].

### Missing data

There were no included studies that reported on the following: CVD hospitalization rates, the use of individual components of triple therapy, CVD investigations or interventions. Full details of hospitalization data extracted from all records assessed for quality can be found in Additional file 2: Table S4.

## Discussion

To the best of our knowledge, this is the first systematic review that has explored the epidemiology and management of CVD by Pacific-specific ethnicity or place of birth. The scant attention to this topic is remarkable given the documented challenges of LMIC in preventing premature deaths related to CVD [24], and the emigration of Pacific people to countries such as USA, NZ, and Australia, which started decades ago.

Data from the included texts showed substantial differences in the mortality, incidence, prevalence and management of CVD (composite, by type) by Pacific country and/or Pacific ethnic group.

Differences in CVD mortality (by type) were as large as 20 times higher between Pacific countries, which was recorded for hypertensive heart disease between Palau and the Cooks Islands [3], and for IHD between Kiribati and the Marshall Islands [21]. Differences in CVD prevalence (by type) were as large as 9 times higher between Pacific countries, which was recorded between Palau and the Cook Islands (also recorded for hypertensive heart disease) and differences in CVD incidence (by type) were as large as 3 times higher, as seen between Guam and Kiribati (recorded for intracerebral haemorrhage) [3]. Differences in the management of CVD were as large as four-fold between the Solomon Islands and Tuvalu [23].

Differences in the mortality (composite) and prevalence (by type) were reported between Pacific ethnic groups living in NZ. The largest difference in CVD (composite) mortality was reported as almost double for Cook Islands Māori than in Tongan people in NZ [1]. The prevalence of IHD, PVD, and heart failure was highest in Cook Islands Māori people in NZ, and 1.3 times higher than in Niuean, 1.4 times higher than in Samoan, and 1.7 times higher than in Niuean peoples respectively [2]. The prevalence of stroke was 1.4 times higher in Samoan than in Tongan people living in NZ [2].

These results illustrate the danger when seeking to understand the burden of CVD for Pacific people as a homogenised group. There is rich diversity among Pacific peoples that spreads beyond ethnicity and includes differing migration patterns and citizenship status, particularly in NZ [1, 9, 25]. Pacific people from the Cook Islands and Niue have been NZ citizens since 1901, while Tokelauans have been NZ citizens since 1916, and Western Samoa a territory under NZ administration until 1962 [1, 26]. Pacific people from other Pacific Island nations, including Tonga and Fiji, have experienced different and shorter migration histories [1, 9, 25], suggesting that longer duration in NZ may relate to higher CVD mortality and prevalence. There is also the possibility of the ‘healthy migrant effect’, where people with better health status are more likely to migrate than those suffering ill-health. However, previous research has suggested that the ‘healthy migrant effect’ may not apply to Pacific people migrating to NZ [9].

### Limitations of the evidence

There were a number of limitations related to the evidence included in the review. Firstly, for the management of CVD it was unclear which medications were considered for managing CVD risk [23]. Secondly, there may be differences in the recording and reporting of health data and classification of cause of death that may explain some of these differences which contribute to interoperability concerns in the Pacific region [27], and may over- or underestimate the differences in prevalence and mortality attributed to CVD. For example, there was a difference in mortality rate due to hypertensive heart disease being 20 times higher in the Cook Islands than in Palau, yet, over the ten year period of 2009 to 2019, IHD remained the leading cause of death in Palau (preceding diabetes) and the second leading cause of death in the Cook Islands (preceded by diabetes) [28, 29]. Hypertensive heart disease was recorded as the fourth leading cause of death in the Cook Islands but 75th in Palau [28, 29], despite its clear relationship to IHD.

It is important to note that the GBD study makes many adjustments and imputations when deriving the country estimates, often based on very limited primary data. This is particularly common among LMICs, of which 10 of the 17 Pacific Island countries whose data were included in this review are [30–33]. In contrast, other studies are more likely to have used primary data. So what at first glance may look like reasonably precisely enumerated estimates in the GBD study could be considerably influenced by imputations for missing data [31].

Ethnicity was re-classified differently between the two texts that reported on the burden of CVD in Pacific-specific groups in NZ [1, 2]. In the metro-Auckland population of people who had identified as Pacific in 2011, Pacific-specific ethnic groups were assigned in reverse population size order in order to attain the largest subgroup sizes [2]. Niuean ethnicity was prioritised (if Niue had been selected as their ethnicity on their health contact record for 2011), followed by Cook Islands Māori (if present in 2011 health contact record), then Tongan (if present in 2011 health contact record), then Samoan (if present in 2011 health contact record) [2]. Other Pacific was assigned if none of the previous Pacific-specific ethnic groups were present [2]. This differed from the total response (overlapping) count used in the other text, which allowed for each person to be assigned to each of the ethnic groups they reported [1]. This is considered beneficial for representing people in each ethnic group they identify with, but presents complexities with making comparisons between groups, as people can be assigned to multiple groups [34]. The lack of consistency in NZ analyses of defining Pacific-specific ethnicity is problematic for tracking the burden of CVD over time.

### Limitations of review processes

There are several limitations of this review which need to be considered when interpreting findings. In terms of the nature of the review, we assumed that data reported by Pacific Island country (excluding Fiji) represented homogenous indigenous populations. It is possible that Pacific countries, particularly those which have been or are currently territories of USA or France do not have a homogenous indigenous Pacific population anymore. Combining data from indigenous and non-indigenous populations may have masked the true size of the effect for indigenous Pacific populations in these countries.

In terms of the studies identified, there are a number of notable observations. First, the literature on this topic was sparse. The limited evidence are worrying [1] for the South-Pacific region that is seeing an increase in the burden of non-communicable diseases such as CVD [35], and [2] because the Pacific diaspora now outnumber the population of many Pacific countries but experience inequities in exposure to CVD risk factors in their new home countries [36]. Second, none of the named first authors of the 23 texts for which quality was assessed were (to our knowledge) of indigenous Pacific ethnicity (see Table 2). A likely implication of this is that the data collection, interpretation and/or reporting is likely not representative of indigenous Pacific worldviews and perspectives. In contrast, the first and last author of this study are each of indigenous Pacific ethnicity. Third, there were important variations between studies in the age of participants, methods and data collected (which is why we were unable to undertake a meta-analysis).

**Table 2:** Pacific group reported, country of first author, by citation

Citation	Pacific group reported. Country (international), Ethnicity (New Zealand, Australia)	Country of first author (if known)
Bennett 1984	Cook Islands, Niue	England, USA
Blakely 2009	In NZ: Samoan, Cook Islands Māori, Tongan, Niuean	New Zealand
Crews 1982	American Samoa	USA
GBD 2019	American Samoa, Cook Islands, Guam, Kiribati, Marshall Islands, Federated States of Micronesia, Nauru, Niue, Northern Mariana Islands, Palau, Papua New Guinea, Samoa, Solomon Islands, Tokelau, Tonga, Tuvalu, Vanuatu	N/A
HPCG 2012	In NZ: Samoan, Tongan, Cook Islands Māori, Niuean	New Zealand
Ichiho 2013	Federated States of Micronesia: State of Yap	USA
Ichiho 2013	Federated States of Micronesia: State of Chuuk	USA
Ichiho 2013	Marshall Islands: Kwajelein Atoll	USA
Ichiho 2013	American Samoa	USA
Ichiho 2013	Northern Mariana Islands	USA
Ichiho 2013	Guam	USA
Ichiho 2013	Palau	USA
Ichiho 2013	Marshall Islands: Majuro Atoll	USA
Ichiho 2013	Federated States of Micronesia: State of Pohnpei	USA
Li 1994	Fiji, Nauru, Kiribati, Cook Islands, Niue, Western Samoa, New Caledonia	Finland
Mendis 2011	Global	Sri Lanka
Novak 2007	In NZ: Samoan, Tongan, Cook Islands Māori, Niuean	New Zealand
Queensland Health 2010	In Australia: Samoan	Australia
Queensland Health 2011	In Australia: Papua New Guinean	Australia
Reed 1970	Chamorro in Rota, Guam, California USA	USA
Taylor 1987	Wallisians in Wallis and New Caledonia	Australia
WHO 2014	Global	Sri Lanka
WHO 2018	Global	N/A

Despite the strategies that were used to ensure its completeness, this review could have missed relevant data.

### Implications

Health and mortality data reporting is limited across the Pacific [37]. Of the 25 Pacific countries listed, data were available for only 17 of those countries in this review. This may reflect delays in implementation of critical infrastructure [38], such as recording cause of death, which has only been recorded in French Polynesia since 1984 [39]. In addition to this, a notable observation was the reporting of diabetes as a leading cause of mortality in Pacific countries [12, 13, 18, 20, 28, 29]. Differences in diabetes mortality between countries were reported as being over eight times higher in Kiribati (203.99 (158.28, 252.68)) than in Guam (23.70 (19.89, 28.63)) [3]. We did not specifically search for diabetes as a cause of mortality as we instead considered it to be a risk factor for developing CVD as a complication [40]. There may be inconsistencies in the way mortality associated with diabetes is recorded. This, along with the limited reporting of health and mortality data in the Pacific, will need further exploration, which is outside the scope of this paper.

Given the differences in burden of CVD evident in the literature, the homogenised approach to understanding the epidemiology of CVD among Pacific peoples, that is evident in the USA, NZ, and Australia where most Pacific people migrate to, may not be appropriate. Exploration of Pacific cardiovascular epidemiology and management by Pacific-specific ethnicity, location, and place of birth is urgently needed to better understand the diversity of CVD health needs and hence health service requirements of Pacific peoples.

## Conclusions

This review of published studies reveals that the epidemiology of CVD among Pacific people varies by specific ethnic groups, place of birth, and country of residence. There is a critical need for high-quality contemporary ethnic-specific Pacific data to respond to the diverse CVD health needs in these underrepresented groups.

## Supplementary Information

**Additional file 1:** Search strategy. Figure 1 shows the search strategy used for MEDLINE (OVID). Figure 2 shows the search strategy used for EMBASE. Figure 3 shows the search strategy used for Scopus. Figure 4 shows the search strategy used for PubMed. Figure 5 shows the search strategy used for the Cochrane Database of Systematic Reviews. As relevant studies and systematic reviews were identified they were checked for additional relevant cited and citing articles / systematic reviews. Figure 6 shows the websites of the World Health Organization, South Pacific Commission, and New Zealand and Pacific governments and health organisations that were searched to identify relevant reports (published up to 20 November 2020).

**Additional file 2:** Study characteristics, NHLBI quality assessment details, and results tables. Description of data: Table 1 shows the characteristics of texts where quality was assessed (n = 23). Table 2 presents the NHLBI quality assessment of the 23 assessed records. Table 3 shows the CVD mortality (composite, by type) outcomes reported by all texts assessed for quality. Table 4 shows CVD hospitalisations outcomes reported by all texts assessed for quality. Table 5 presents the CVD incidence/prevalence (composite) outcomes reported by all texts assessed for quality. Table 6 presents the CVD mortality (composite, by type) outcomes reported by all texts assessed for quality. Tables 7–8 show the management outcomes reported by all texts assessed for quality.

## Data Availability

Not applicable.

## Abbreviations

AF: Atrial fibrillation
CABG: Coronary artery bypass grafts
CeVD: Cerebrovascular disease
CI: Confidence interval
CVD: Cardiovascular disease(s)
GBD: Global Burden of Disease
HPCG: Health Partners Consulting Group
IHD: Ischemic heart disease
LMIC: Low- and middle-income countries
MESH: Medical subject headings
NHLBI: National Heart, Lung, and Blood Institute
NZ: Aotearoa New Zealand
PCI: Percutaneous coronary intervention
PNG: Papua New Guinea
PRISMA: Preferred Reporting Items for Systematic Reviews and Meta-Analyses.
PVD: Peripheral vascular disease
USA: United States of America
WHO: World Health Organization
