# Supplementary material for: Cardiovascular disease and its management among Pacific people: a systematic review by ethnicity and place of birth
Source: BMC Cardiovasc Disord. 2021 Oct 24;21:515. doi: 10.1186/s12872-021-02313-x (PMC8543825; doi:10.1186/s12872-021-02313-x)
Supplement: Supplementary file 1 — Additional file 1: Search strategy. Figure 1 shows the search strategy used for MEDLINE (OVID). Figure 2 shows the search strategy used for EMBASE. Figure 3 shows the search strategy used for Scopus. Figure 4 shows the search strategy used for PubMed. Figure 5 shows the search strategy used for the Cochrane Database of Systematic Reviews. As relevant studies and systematic reviews were identified they were checked for additional relevant cited and citing articles / systematic reviews. Figure 6 shows the websites of the World Health Organization, South Pacific Commission, and New Zealand and Pacific governments and health organisations that were searched to identify relevant reports (published up to 20 November 2020). [file 12872_2021_2313_MOESM1_ESM.docx]

Additional File 1: Search Strategy

## Figure 1: Search terms and strategy for MEDLINE (OVID)

| 1 | exp Myocardial Infarction/ |
| --- | --- |
| 2 | myocardial infarction.mp. |
| 3 | Myocardial ischemia.mp. or exp Myocardial Ischemia/ |
| 4 | exp Ischemic Attack, Transient/ |
| 5 | Ischemic attack.mp. |
| 6 | exp Stroke/ |
| 7 | Stroke.mp. |
| 8 | peripheral vascular diseases/ or exp peripheral arterial disease/ |
| 9 | peripheral vascular disease.mp. |
| 10 | peripheral arterial disease.mp. |
| 11 | exp Arteriosclerosis/ |
| 12 | arteriosclero*.mp. |
| 13 | exp Heart Failure/ |
| 14 | heart failure.mp. |
| 15 | exp Atrial Fibrillation/ |
| 16 | atrial fibrillation.mp. |
| 17 | exp incidence/ or exp prevalence/ or exp mortality/ |
| 18 | (incidence or prevalence or mortality).mp. |
| 19 | exp Hospitalization/ |
| 20 | hospitalization.mp. |
| 21 | exp "Treatment Adherence and Compliance"/ |
| 22 | (Treatment Adherence and Compliance).mp. |
| 23 | Treatment adherence.mp. |
| 24 | Treatment compliance.mp. |
| 25 | exp Patient Care Management/ |
| 26 | Patient Care Management.mp. |
| 27 | exp Health Services Accessibility/ |
| 28 | Health Services Accessibility.mp. |
| 29 | Health care access.mp. |
| 30 | exp "quality of health care"/ or exp "health care quality, access, and evaluation"/ |
| 31 | (health care quality, access, and evaluation).mp. |
| 32 | quality of health care.mp. |
| 33 | Epidemiology/ |
| 34 | epidemiology.mp. |
| 35 | Availability of health services.mp. |
| 36 | Pacific people.mp. |
| 37 | pacific islands/ or exp melanesia/ or exp micronesia/ or exp polynesia/ |
| 38 | Pacific island*.mp. |
| 39 | melanesia.mp. |
| 40 | micronesia.mp. |
| 41 | polynesia.mp. |
| 42 | Pacifica.mp. |
| 43 | Pasifika.mp. |
| 44 | Pasefika.mp. |
| 45 | Pan-Pacific.mp. |
| 46 | Niue*.mp. |
| 47 | Cook Islander.mp. |
| 48 | Cook Island Maori.mp. |
| 49 | Fiji*.mp. |
| 50 | Native Hawai*.mp. |
| 51 | Tokelau*.mp. |
| 52 | Kiribati.mp. |
| 53 | exp Oceanic Ancestry Group/ |
| 54 | Oceanic Ancestry Group.mp. |
| 55 | Kanaka Maoli.mp. |
| 56 | Tuvaluan.mp. |
| 57 | Tahitian.mp. |
| 58 | Nauruan.mp. |
| 59 | Futunan.mp. |
| 60 | “Papua New Guinea".mp. or Papua New Guinea/ |
| 61 | Solomon Island*.mp. |
| 62 | Vanuatuan.mp. |
| 63 | "French Polynesia".mp. |
| 64 | Tonga*.mp. |
| 65 | New Caledonia*.mp. |
| 66 | Pohnpei*.mp. |
| 67 | Chamorro*.mp. |
| 68 | Guamanian*.mp. |
| 69 | Palau*.mp. |
| 70 | Hawai*.mp. |
| 71 | Pitcairn Island*.mp. |
| 72 | Samoa*.mp. |
| 73 | Rotuma*.mp. |
| 74 | marshallese.mp. |
| 75 | easter island*.mp. |
| 76 | Rarotonga*.mp. |
| 77 | Pacific area.mp. |
| 78 | Islands of the Pacific.mp. |
| 79 | Moana.mp. |
| 80 | New Hebrides.mp. |
| 81 | Mariana Island*.mp. |
| 82 | Caroline Island*.mp. |
| 83 | Marquesas.mp. |
| 84 | Ellice island*.mp. |
| 85 | 1 or 2 or 3 or 4 or 5 or 6 or 7 or 8 or 9 or 10 or 11 or 12 or 13 or 14 or 15 or 16 |
| 86 | 17 or 18 or 19 or 20 or 21 or 22 or 23 or 24 or 25 or 26 or 27 or 28 or 29 or 30 or 31 or 32 or 33 or 34 or 35 |
| 87 | 36 or 37 or 38 or 39 or 40 or 41 or 42 or 43 or 44 or 45 or 46 or 47 or 48 or 49 or 50 or 51 or 52 or 53 or 54 or 55 or 56 or 57 or 58 or 59 or 60 or 61 or 62 or 63 or 64 or 65 or 66 or 67 or 68 or 69 or 70 or 71 or 72 or 73 or 74 or 75 or 76 or 77 or 78 or 79 or 80 or 81 or 82 or 83 or 84 |
| 88 | 85 and 86 and 87 |
| 89 | limit 88 to "humans only (removes records about animals)" |

## Figure 2: Search terms and strategy for EMBASE

| 1 | exp incidence/ or incidence.mp. |
| --- | --- |
| 2 | prevalence.mp. or exp prevalence/ |
| 3 | premature mortality/ or mortality.mp. or exp mortality/ or cardiovascular mortality/ |
| 4 | hospitalization.mp. or exp hospitalization/ |
| 5 | epidemiology/ or epidemiology.mp. |
| 6 | treatment adherence.mp. or exp patient compliance/ |
| 7 | treatment compliance.mp. |
| 8 | patient care management.mp. or exp patient care/ |
| 9 | health services accessibility.mp. or exp health care delivery/ |
| 10 | health care access.mp. |
| 11 | quality of health care.mp. or exp health care quality/ |
| 12 | exp health care availability/ or availability of health services.mp. |
| 13 | 1 or 2 or 3 or 4 or 5 or 6 or 7 or 8 or 9 or 10 or 11 or 12 |
| 14 | myocardial infarction.mp. or exp heart infarction/ |
| 15 | myocardial ischemia.mp. or exp heart muscle ischemia/ |
| 16 | transient ischemic attack.mp. or exp transient ischemic attack |
| 17 | ischemic attack.mp. |
| 18 | stroke.mp. or exp cerebrovascular accident/ |
| 19 | peripheral vascular disease.mp. or exp peripheral vascular disease/ |
| 20 | peripheral arterial disease.mp. or exp peripheral occlusive artery disease/ |
| 21 | exp atherosclerosis/ or exp arteriosclerosis/ or arteriosclero*.mp. |
| 22 | heart failure.mp. or exp heart failure/ |
| 23 | atrial fibrillation.mp. or exp atrial fibrillation/ |
| 24 | 14 or 15 or 16 or 17 or 18 or 19 or 20 or 21 or 22 or 23 |
| 25 | Pacific people.mp. |
| 26 | Pacific islands/ or Pacific island*.mp. |
| 27 | melanesia*.mp. or exp Melanesia/ |
| 28 | exp "Federated States of Micronesia"/ or micronesia*.mp. |
| 29 | polynesia*.mp. or exp Polynesia/ |
| 30 | Pacifica.mp. |
| 31 | Pasifika.mp. |
| 32 | Pasefika.mp. |
| 33 | Pan-Pacific.mp. |
| 34 | exp Niue/ or Niue*.mp. |
| 35 | Cook Islander*.mp. or exp Cook Islander/ |
| 36 | 036 Cook Island Maori.mp. |
| 37 | exp Fiji/ or Fiji*.mp. |
| 38 | exp Native Hawaiian/ or Native Hawai*.mp. |
| 39 | exp Tokelau/ or Tokelau*.mp. |
| 40 | Kiribati.mp. or exp Kiribati/ |
| 41 | Oceanic ancestry group.mp. or exp Oceanic ancestry group/ |
| 42 | Kanaka Maoli.mp. |
| 43 | exp Tuvalu/ or tuvalu*.mp. |
| 44 | exp Nauru/ or Nauru*.mp. |
| 45 | exp Tahiti/ or Tahiti*.mp. |
| 46 | exp "Wallis and Futuna"/ or Futuna*.mp. |
| 47 | exp Papua New Guinea/ or Papua New Guinea*.mp. |
| 48 | exp Solomon Islands/ or Solomon Island*.mp. |
| 49 | exp Vanuatu/ or Vanuatu*.mp. |
| 50 | exp French Polynesia/ or French Polynesia*.mp. |
| 51 | exp Tonga/ or Tonga*.mp. |
| 52 | exp New Caledonia/ or New Caledonia*.mp. |
| 53 | Pohnpei*.mp. or exp Pohnpei State/ |
| 54 | exp "Chamorro (people)"/ or Chamorro*.mp. |
| 55 | exp Guam/ or Guam*.mp. |
| 56 | exp Palau/ or Palau*.mp. |
| 57 | exp Hawaii/ or Hawai*.mp. |
| 58 | exp Pitcairn/ or Pitcairn Island*.mp. |
| 59 | exp Samoa/ or exp American Samoa/ or Samoa*.mp. |
| 60 | Rotuma*.mp. |
| 61 | Marshallese.mp. or exp Marshallese/ |
| 62 | Easter Island*.mp. |
| 63 | Rarotonga*.mp. |
| 64 | Pacific area.mp. |
| 65 | Islands of the Pacific.mp. |
| 66 | Moana.mp. |
| 67 | New Hebrides.mp. |
| 68 | exp Northern Mariana Islands/ or Mariana island*.mp. |
| 69 | Caroline island*.mp. |
| 70 | Marquesas.mp. |
| 71 | Ellice island*.mp. |
| 72 | 25 or 26 or 27 or 28 or 29 or 30 or 31 or 32 or 33 or 34 or 35 or 36 or 37 or 38 or 39 or 40 or 41 or 42 or 43 or 44 or 45 or 46 or 47 or 48 or 49 or 50 or 51 or 52 or 53 or 54 or 55 or 56 or 57 or 58 or 59 or 60 or 61 or 62 or 63 or 64 or 65 or 66 or 67 or 68 or 69 or 70 or 71 |
| 73 | 13 and 24 and 72 |
| 74 | limit 73 to "humans only (removes records about animals)" |

## Figure 3: Search terms and strategy for Scopus

| TITLE-ABS-KEY ( epidemiolog*  OR  inciden*  OR  prevalen*  OR  mortalit*  OR  hospitaliz*  OR  management  OR  "treatment adherence"  OR  "treatment compliance"  OR  "Patient care management"  OR  "Patient care"  OR  "health services access*"  OR  "health care delivery"  OR  "health care access"  OR  access*  OR  "Quality of health care"  OR  "health care quality"  OR  quality  OR  evaluat*  OR  "health care availability"  OR  "availability of health service" )  AND  TITLE-ABS-KEY ( "Cardiovascular disease*"  OR  "Myocardial Ischemia"  OR  "Myocardial Infarction"  OR  ischemi*  OR  "heart infarction"  OR  "transient ischemic attack"  OR  "ischemic attack"  OR  stroke  OR  "Peripheral vascular disease"  OR  arteriosclero*  OR  "Atherosclero*"  OR  "Peripheral arterial disease"  OR  "Heart Failure"  OR  "Atrial Fibrillation" )  AND  TITLE-ABS-KEY ( "Pacific people"  OR  "Pacific island*"  OR  melanesia*  OR  micronesia*  OR  polynesia*  OR  pacifica  OR  pasifika  OR  pasefika  OR  pan-pacific  OR  niue*  OR  "Cook Island*"  OR  fiji*  OR  "Native Hawai*"  OR  tokelau*  OR  kiribati  OR  oceani*  OR  "Kanaka Maoli"  OR  tuvalu*  OR  tahiti*  OR  nauru*  OR  futuna*  OR  "Papua New Guinea*"  OR  "Solomon Island*"  OR  vanuatu*  OR  "French Polynesia*"  OR  tonga*  OR  "New Caledonia*"  OR  pohnpei*  OR  chamorro*  OR  guam*  OR  palau*  OR  hawai*  OR  "Pitcairn Island*"  OR  samoa*  OR  rotuma*  OR  marshall*  OR  "Easter Island*"  OR  rarotonga*  OR  "Pacific area"  OR  "Islands of the Pacific"  OR  moana  OR  "New Hebrides"  OR  "Mariana Island*"  OR  "Caroline Island*"  OR  marquesas  OR  "Ellice Island*"  OR  pasifiki ) |
| --- |

## Figure 4: Search terms and strategy for PubMed

| ((((((((((((((((((((("treatment adherence and compliance"[MeSH Terms])) OR "patient care management"[MeSH Terms]) OR "health services accessibility"[MeSH Terms]) OR ("health care quality, access, and evaluation"[MeSH Terms])) OR "quality of health care"[MeSH Terms]) OR ("treatment adherence[Text Word] AND compliance"[Text Word])) OR "treatment adherence"[Text Word]) OR "treatment compliance"[Text Word]) OR "patient care management"[Text Word]) OR "health services accessibility"[Text Word]) OR "health care access"[Text Word]) OR "health care quality"[Text Word]) OR "health care evaluation"[Text Word]) OR "quality of health care"[Text Word]) OR "availability of health services"[Text Word])) OR (((((((((((("incidence"[MeSH Terms]) OR "prevalence"[MeSH Terms]) OR "mortality"[MeSH Terms]) OR "hospitalization"[MeSH Terms]) OR "epidemiology"[MeSH Terms]) OR incidence[Text Word]) OR prevalence[Text Word]) OR mortality[Text Word]) OR hospitalization[Text Word]) OR hospitalisation[Text Word]) OR epidemiology) AND Humans[Mesh])) AND Humans[Mesh])) OR (((((((((((((((((("myocardial infarction"[MeSH Terms]) OR "myocardial ischemia"[MeSH Terms]) OR "ischemic attack, transient"[MeSH Terms]) OR "stroke"[MeSH Terms]) OR "peripheral vascular diseases"[MeSH Terms]) OR "peripheral arterial disease"[MeSH Terms]) OR "arteriosclerosis"[MeSH Terms]) OR "heart failure"[MeSH Terms]) OR "atrial fibrillation"[MeSH Terms]) OR "myocardial infarction"[Text Word]) OR "myocardial ischemia"[Text Word]) OR "ischemic attack"[Text Word]) OR stroke[Text Word]) OR "peripheral vascular disease"[Text Word]) OR "peripheral arterial disease"[Text Word]) OR "arteriosclero*"[Text Word]) OR "heart failure"[Text Word]) OR "atrial fibrillation"[Text Word])) OR ((((((((((((((((((((((((((((((((((((((((((((((((((("pacific islands"[MeSH Terms]) OR "melanesia"[MeSH Terms]) OR "micronesia"[MeSH Terms]) OR "polynesia"[MeSH Terms]) OR "oceanic ancestry group"[MeSH Terms]) OR "Pacific people"[Text Word]) OR "Pacific island*"[Text Word]) OR Melanesia[Text Word]) OR Micronesia[Text Word]) OR Polynesia[Text Word]) OR Pacifica[Text Word]) OR Pasifika[Text Word]) OR Pasefika[Text Word]) OR "Pan-Pacific"[Text Word]) OR Niue*[Text Word]) OR "Cook Island*"[Text Word]) OR Fiji*[Text Word]) OR "Native Hawai*"[Text Word]) OR Tokelau*[Text Word]) OR Kiribati[Text Word]) OR "Oceanic ancestry group"[Text Word]) OR "Kanaka Maoli"[Text Word]) OR Tuvaluan[Text Word]) OR Tahitian[Text Word]) OR Nauruan[Text Word]) OR Futunan[Text Word]) OR "Papua New Guinea"[Text Word]) OR "Solomon Island*"[Text Word]) OR Vanuatuan[Text Word]) OR "French Polynesia*"[Text Word]) OR Tonga*[Text Word]) OR "New Caledonia*"[Text Word]) OR Pohnpei*[Text Word]) OR Chamorro*[Text Word]) OR Guamanian[Text Word]) OR Palau*[Text Word]) OR Hawai*[Text Word]) OR "Pitcairn Island*"[Text Word]) OR Samoa*[Text Word]) OR Rotuma*[Text Word]) OR Marshallese[Text Word]) OR "Easter Island*"[Text Word]) OR Rarotonga*[Text Word]) OR "Pacific area"[Text Word]) OR "Islands of the Pacific"[Text Word]) OR Moana[Text Word]) OR "New Hebrides"[Text Word]) OR "Mariana Island*"[Text Word]) OR "Caroline Island*"[Text Word]) OR Marquesas[Text Word]) OR "Ellice Island*"[Text Word]) |
| --- |
| Filters: Humans |

## Figure 5: Search terms and strategy for Cochrane Library

| ID | Search |
| --- | --- |
| #1 | MeSH descriptor: [Incidence] explode all trees |
| #2 | ("incidence rate"):ti,ab,kw (Word variations have been searched) |
| #3 | MeSH descriptor: [Prevalence] explode all trees |
| #4 | (prevalence):ti,ab,kw (Word variations have been searched) |
| #5 | MeSH descriptor: [Mortality] explode all trees |
| #6 | (mortality):ti,ab,kw (Word variations have been searched) |
| #7 | MeSH descriptor: [Hospitalization] explode all trees |
| #8 | (hospitalization):ti,ab,kw (Word variations have been searched) |
| #9 | MeSH descriptor: [Epidemiology] this term only |
| #10 | (epidemiology):ti,ab,kw (Word variations have been searched) |
| #11 | MeSH descriptor: [Treatment Adherence and Compliance] explode all trees |
| #12 | ("Treatment adherence and compliance"):ti,ab,kw (Word variations have been searched) |
| #13 | ("treatment adherence"):ti,ab,kw (Word variations have been searched) |
| #14 | ("treatment compliance"):ti,ab,kw (Word variations have been searched) |
| #15 | ("patient care management"):ti,ab,kw (Word variations have been searched) |
| #16 | MeSH descriptor: [Patient Care Management] explode all trees |
| #17 | MeSH descriptor: [Health Services Accessibility] explode all trees |
| #18 | ("Health services accessibility"):ti,ab,kw (Word variations have been searched) |
| #19 | ("Health care access"):ti,ab,kw (Word variations have been searched) |
| #20 | MeSH descriptor: [Quality of Health Care] explode all trees |
| #21 | MeSH descriptor: [Health Care Quality, Access, and Evaluation] explode all trees |
| #22 | ("Health care quality, access, and evaluation"):ti,ab,kw (Word variations have been searched) |
| #23 | ("Quality of health care"):ti,ab,kw (Word variations have been searched) |
| #24 | ("Availability of health services"):ti,ab,kw (Word variations have been searched) |
| #25 | (#1 OR #2 OR #3 OR #4 OR #5 OR #6 OR #7 OR #8 OR #9 OR #10 OR #11 OR #12 OR #13 OR #14 OR #15 OR #16 OR #17 OR #18 OR #19 OR #20 OR #21 OR #22 OR #23 OR #24) |
| #26 | MeSH descriptor: [Myocardial Infarction] explode all trees |
| #27 | ("Myocardial infarction"):ti,ab,kw (Word variations have been searched) |
| #28 | MeSH descriptor: [Myocardial Ischemia] explode all trees |
| #29 | ("myocardial ischemia"):ti,ab,kw (Word variations have been searched) |
| #30 | MeSH descriptor: [Ischemic Attack, Transient] explode all trees |
| #31 | ("ischemic attack"):ti,ab,kw (Word variations have been searched) |
| #32 | MeSH descriptor: [Stroke] explode all trees |
| #33 | (stroke):ti,ab,kw (Word variations have been searched) |
| #34 | MeSH descriptor: [Peripheral Vascular Diseases] explode all trees |
| #35 | MeSH descriptor: [Peripheral Arterial Disease] explode all trees |
| #36 | ("peripheral vascular disease"):ti,ab,kw (Word variations have been searched) |
| #37 | ("peripheral arterial disease"):ti,ab,kw (Word variations have been searched) |
| #38 | MeSH descriptor: [Arteriosclerosis] explode all trees |
| #39 | (arteriosclero*):ti,ab,kw (Word variations have been searched) |
| #40 | MeSH descriptor: [Heart Failure] explode all trees |
| #41 | ("heart failure"):ti,ab,kw (Word variations have been searched) |
| #42 | MeSH descriptor: [Atrial Fibrillation] explode all trees |
| #43 | ("atrial fibrillation"):ti,ab,kw (Word variations have been searched) |
| #44 | #26 OR #27 OR #28 OR #29 OR #30 OR #31 OR #32 OR #33 OR #34 OR #35 OR #36 OR #37 OR #38 OR #39 OR #40 OR #41 OR #42 OR #43 |
| #45 | MeSH descriptor: [Melanesia] explode all trees |
| #46 | MeSH descriptor: [Polynesia] explode all trees |
| #47 | MeSH descriptor: [Micronesia] explode all trees |
| #48 | MeSH descriptor: [Micronesia] explode all trees |
| #49 | MeSH descriptor: [Oceanic Ancestry Group] explode all trees |
| #50 | ("Pacific people"):ti,ab,kw (Word variations have been searched) |
| #51 | ("Pacific island*"):ti,ab,kw (Word variations have been searched) |
| #52 | ("Melanesia"):ti,ab,kw (Word variations have been searched) |
| #53 | ("Polynesia"):ti,ab,kw (Word variations have been searched) |
| #54 | ("Micronesia"):ti,ab,kw (Word variations have been searched) |
| #55 | ("Pacifica"):ti,ab,kw (Word variations have been searched) |
| #56 | ("Pasifika"):ti,ab,kw (Word variations have been searched) |
| #57 | ("Pasefika"):ti,ab,kw (Word variations have been searched) |
| #58 | ("Pan-Pacific"):ti,ab,kw (Word variations have been searched) |
| #59 | ("Niue*"):ti,ab,kw (Word variations have been searched) |
| #60 | ("Cook Island*"):ti,ab,kw (Word variations have been searched) |
| #61 | ("Fiji*"):ti,ab,kw (Word variations have been searched) |
| #62 | ((Native Hawai*)):ti,ab,kw (Word variations have been searched) |
| #63 | ("Hawai*"):ti,ab,kw (Word variations have been searched) |
| #64 | ("Tokelau*"):ti,ab,kw (Word variations have been searched) |
| #65 | ("Kiribati"):ti,ab,kw (Word variations have been searched) |
| #66 | ("Oceanic ancestry group"):ti,ab,kw (Word variations have been searched) |
| #67 | ("Kanaka Maoli"):ti,ab,kw (Word variations have been searched) |
| #68 | ("Tuvalu*"):ti,ab,kw (Word variations have been searched) |
| #69 | ("Nauru*"):ti,ab,kw (Word variations have been searched) |
| #70 | ("Futuna*"):ti,ab,kw (Word variations have been searched) |
| #71 | ("Papua New Guinea*"):ti,ab,kw (Word variations have been searched) |
| #72 | ("Solomon Island*"):ti,ab,kw (Word variations have been searched) |
| #73 | ("Vanuatu*"):ti,ab,kw (Word variations have been searched) |
| #74 | ("French Polynesia*"):ti,ab,kw (Word variations have been searched) |
| #75 | ("tonga"):ti,ab,kw (Word variations have been searched) |
| #76 | ("New Caledonia*"):ti,ab,kw (Word variations have been searched) |
| #77 | ("Pohnpei*"):ti,ab,kw (Word variations have been searched) |
| #78 | ("Chamorro"):ti,ab,kw (Word variations have been searched) |
| #79 | ("Guamanian"):ti,ab,kw (Word variations have been searched) |
| #80 | ("Palau*"):ti,ab,kw (Word variations have been searched) |
| #81 | ("Pitcairn Island"):ti,ab,kw (Word variations have been searched) |
| #82 | ("Samoa*"):ti,ab,kw (Word variations have been searched) |
| #83 | ("Rotuma*"):ti,ab,kw (Word variations have been searched) |
| #84 | ("Marshallese"):ti,ab,kw (Word variations have been searched) |
| #85 | ("Easter Island*"):ti,ab,kw (Word variations have been searched) |
| #86 | ("Rarotonga"):ti,ab,kw (Word variations have been searched) |
| #87 | ("Pacific area"):ti,ab,kw (Word variations have been searched) |
| #88 | ("Islands of the Pacific"):ti,ab,kw (Word variations have been searched) |
| #89 | ("Moana"):ti,ab,kw (Word variations have been searched) |
| #90 | ("New Hebrides"):ti,ab,kw (Word variations have been searched) |
| #91 | ("Mariana Island*"):ti,ab,kw (Word variations have been searched) |
| #92 | ("Caroline Island*"):ti,ab,kw (Word variations have been searched) |
| #93 | ("Marquesas"):ti,ab,kw (Word variations have been searched) |
| #94 | ("Ellice Island*"):ti,ab,kw (Word variations have been searched) |
| #95 | #45 OR #46 OR #47 OR #48 OR #49 OR #50 OR #51 OR #52 OR #53 OR #54 OR #55 OR #56 OR #57 OR #58 OR #59 OR #60 OR #61 OR #62 OR #63 OR #64 OR #65 OR #66 OR #67 OR #68 OR #69 OR #70 OR #71 OR #72 OR #73 OR #74 OR #75 OR #76 OR #77 OR #78 OR #79 OR #80 OR #81 OR #82 OR #83 OR #84 OR #85 OR #86 OR #87 OR #88 OR #89 OR #90 OR #91 OR #92 OR #93 OR #94 |
| #96 | #25 AND #44 AND #95 (Word variations have been searched) |

## Figure 6: Websites searched for relevant grey literature

| **Country/Organisation** | **Link** |
| --- | --- |
| American Samoa | <https://www.americansamoa.gov/government> |
| Australia Ministry of Health | <https://www.health.gov.au/> |
| Cook Islands | <http://www.ck/govt.htm> |
| Easter Island | <https://www.gob.cl/en/ministries/ministry-of-health/> |
| Federated States of Micronesia | <https://www.fsmgov.org/> |
| Fiji | <https://www.fiji.gov.fj/Home> |
| French Polynesia | <https://www.presidence.pf/> |
| Guam | <https://www.guam.gov/> |
| Hawaii state | <https://portal.ehawaii.gov/> |
| Kiribati | <http://www.president.gov.ki/> |
| Marshall Islands | <https://rmiparliament.org/cms/> |
| Nauru | <http://www.naurugov.nr/> |
| New Caledonia | <https://gouv.nc/> |
| New Zealand Ministry of Health | <https://www.health.govt.nz/>  https://www.researchreview.co.nz/nz/Clinical-Area/Other-Health/Pacific-Health.aspx?Show=RR-All |
| Niue | <http://www.gov.nu/wb/> |
| Palau | <https://www.palaugov.pw/executive-branch/ministries/health/> |
| Papua New Guinea | <https://www.health.gov.pg/> |
| Pitcairn Island | <http://www.government.pn/> |
| Pohnpei | <https://pohnpeistate.gov.fm/> |
| Solomon Islands | <http://www.parliament.gov.sb/> |
| Tokelau | <https://www.tokelau.org.nz/> |
| Tonga | <http://www.health.gov.to/> |
| Tuvalu | <http://www.tuvaluislands.com/gov_addresses.htm> |
| Vanuatu | <https://www.gov.vu/en/> |
| Wallis and Futuna | <http://www.wallis-et-futuna.gouv.fr/> |
| Western Samoa | <https://www.health.gov.ws/> |
| South Pacific Commission | <https://www.spc.int/> |
| WHO | <https://www.who.int/> |

A record of the date and number of relevant reports identified was maintained.
