# Supplementary material for: Cardiovascular disease and its management among Pacific people: a systematic review by ethnicity and place of birth
Source: BMC Cardiovasc Disord. 2021 Oct 24;21:515. doi: 10.1186/s12872-021-02313-x (PMC8543825; doi:10.1186/s12872-021-02313-x)
Supplement: Supplementary file 2 — Additional file 2: Study characteristics, NHLBI quality assessment details, and results tables. Description of data: Table 1 shows the characteristics of texts where quality was assessed (n = 23). Table 2 presents the NHLBI quality assessment of the 23 assessed records. Table 3 shows the CVD mortality (composite, by type) outcomes reported by all texts assessed for quality. Table 4 shows CVD hospitalisations outcomes reported by all texts assessed for quality. Table 5 presents the CVD incidence/prevalence (composite) outcomes reported by all texts assessed for quality. Table 6 presents the CVD mortality (composite, by type) outcomes reported by all texts assessed for quality. Tables 7–8 show the management outcomes reported by all texts assessed for quality. [file 12872_2021_2313_MOESM2_ESM.docx]

Additional File 2: Study characteristics, NHLBI quality assessment details, and results tables

## Table 1: Characteristics of studies and reports where quality was assessed.

| *Citation* | *Location(s)* | *Data Source/Study Population, year(s)* | *Comparison groups* | *NHLBI assessed quality* |
| --- | --- | --- | --- | --- |
| *Records included in systematic review* | | | | |
| Blakely 2009 | New Zealand | New Zealand Census-Mortality Study 2001-2004 | Samoan, Cook Islands Māori, Niuean, Tongan | Good |
| GBD 2019 | Countries listed under Oceania | Global Burden of Disease Study 2019 data resource | American Samoa, Guam, Marshall Islands, Cook Islands, Federated States of Micronesia, Kiribati, Northern Mariana Islands, Papua New Guinea, Nauru, Vanuatu, Niue, Tonga, Palau, Tokelau, Tuvalu, Solomon Islands, Samoa, | Good |
| Health Partners Consulting Group 2012 | Metro-Auckland (Counties Manukau, Auckland, Waitemata), New Zealand | National surveys, the PRIMHD database, clinical network data from the Northern Region DHBs | Samoan, Cook Islands Māori, Tongan, Niuean | Good |
| Mendis 2011 | Global | Global burden of disease estimates | Cook Islands, Kiribati, Marshall Islands, Federated States of Micronesia, Nauru, Niue, Palau, Papua New Guinea, Samoa, Solomon Islands, Tonga, Tuvalu, Vanuatu | Good |
| WHO 2014 | Global | - | Papua New Guinea, Solomon Islands | Good |
| WHO 2018 | Global | Life estimates derived from routinely collected data 2000-2016 | Kiribati, Nauru, Solomon Islands, Tuvalu | Good |
| *Records in which quality was assessed as poor or fair quality* | | | | |
| Bennett 1984 | Niue, Rarotonga | Primary data: Population survey | Niue, Cook Islands (Rarotonga) | Fair |
| Crews 1982 | American Samoa | Official death records from LBJ (Lyndon B. Johnson) Tropical Medical Center 1962-1974 | Areas of residence: modern, intermediate, traditional | Poor |
| Ichiho 2013 | Federated States of Micronesia: State of Yap | Data provided from the Medical Records Office of Yap State Hospital 2008-2010 | * | Poor |
| Ichiho 2013 | Federated States of Micronesia: State of Chuuk | Death certification data obtained from Chuuk State Hospital Medical Records Office 2007-2009 | * | Poor |
| Ichiho 2013 | Marshall Islands: Kwajelein Atoll | Death certification data obtained from Ebeye Hospital. 2007-2009 | * | Poor |
| Ichiho 2013 | American Samoa | American Samoa Statistical Yearbook 2008, 2006-2008 | * | Poor |
| Ichiho 2013 | Northern Mariana Islands | 2008 Health Data Report, 2003-2005 | * | Poor |
| Ichiho 2013 | Guam | Death certificates from the Office of Vital Statistics, Guam Department of Public Health and Social Services, 2003-2005 | * | Poor |
| Ichiho 2013 | Palau | Data provided by epidemiologists at the Bureau of Public Health, 2006-2009 | * | Poor |
| Ichiho 2013 | Marshall Islands: Majuro Atoll | Mortality data reported in Ministry of Health Annual Reports for 2007-2009 | * | Poor |
| Ichiho 2013 | Federated States of Micronesia: Pohnpei | Death certificate data from Pohnpei State Hospital 2007-2009 | * | Poor |
| Li 1994 | Fiji, Nauru, Kiribati, Cook Islands, Niue, Western Samoa, New Caledonia | Primary data: Population survey in 15 population groups 1978-1987 | Cook Islands, Niue, Western Samoa, Fiji (Lakeba), New Caledonia, Fiji (Melanesian), Kiribati, Nauru | Fair |
| Novak 2007 | Counties Manukau, Auckland, New Zealand | Routinely collected national data sources | Samoan, Cook Island, Tongan, Niuean | Fair |
| Queensland Health 2010 | Queensland, Australia | Queensland Hospital Admitted Patient Data Collection, July 2006-June 2008 | Samoan born Queenslanders, total Queenslanders. | Fair |
| Queensland Health 2011 | Queensland, Australia | Queensland Hospital Admitted Patient Data Collection | Papua New Guinean born Queenslanders, total Queenslanders | Fair |
| Reed 1970 | Rota, Guam, California | Primary data: Questionnaire and anthropometric measurements in three samples of Chamorro | Chamorro in Rota, Guam, and California | Poor |
| Taylor 1987 | Wallis Island, Noumea, New Caledonia | Primary data: Survey and anthropometric measurements in two sample populations | Wallisians living in villages on Wallis Island, Wallisians working in or living near a nickel factory in Noumea, New Caledonia | Poor |

*Included because although these studies are reported separately, they were conducted by the same research group, using the same methods and collectively portray a regional perspective where we were able to identify Pacific-specific comparison data.

## Table 2: Application of the NHLBI quality assessment tool to the records assessed for quality.

| Citation | Q1. Research question | Q2. Study population defined | Q3. Study population participation rate | Q4. Recruitment | Q5. Sample size | Q6. Exposure pre outcome | Q7. Time sufficient | Q8. Different exposures level | Q9. Exposure assessment | Q10. Repeated exposure | Q11. Outcome measures | Q12. Blinding | Q13. Follow up | Q14. Statistical analyses | Overall Rating | Additional notes |
| --- | --- | --- | --- | --- | --- | --- | --- | --- | --- | --- | --- | --- | --- | --- | --- | --- |
| *Records included in systematic review* | | | | | | | | | | | | | | | | |
| Blakely 2009 | Y | Y | Y | Y | NA | NA | NA | NA | Y | NA | Y | Y | Y | Y | Good | Ethnicity clearly defined. Rate ratios adjusted by sex, age, country of birth, and income. |
| GBD 2019 | Y | Y | Y | NA | NA | NA | NA | NA | NA | NA | Y | Y | Y | NA | Good | National rates reported, age-standardized rates for mortality, prevalence and incidence. |
| Health Partners Consulting Group 2012 | Y | Y | Y | NA | NA | NA | NA | NA | Y | NA | Y | Y | NA | Y | Good | All outcomes clearly defined and age-standardized. Construction of metro-Auckland Pacific population clearly defined. |
| Mendis 2011 | Y | Y | Y | Y | NA | NA | NA | NA | NA | NA | Y | Y | Y | Y | Good | Outcome clearly defined. Age standardized mortality rates provided. |
| WHO 2014 | Y | Y | Y | Y | NA | NA | NA | NA | NA | NA | Y | Y | Y | Y | Good | Age standardized death rates for cause provided by sex. |
| WHO 2018 | Y | Y | Y | Y | NA | NA | NA | NA | NA | NA | Y | Y | Y | NA | Good | National proportions reported. |
| *Records in which quality was assessed as poor or fair quality* | | | | | | | | | | | | | | | | |
| Bennett 1984 | Y | Y | Y | Y | N | NA | NA | NA | NA | NA | Y | N | NA | Y | Fair | Outcome measures defined. Age-adjusted results reported. No sample size justification, power description, variance, effect estimates. |
| Crews 1982 | Y | Y | NA | Y | NA | NA | NA | NA | Y | NA | Y | Y | Y | N | Poor | Population level study using death certificates (1962-1974). American Samoan Census 1970 used to define average population at risk during the period of 1962-1974. No age adjustment. |
| Ichiho 2013  Pohnpei | Y | Y | Y | Y | NA | NA | NA | NA | NA | NA | N | N | NA | N | Poor | Outcome measures not clearly defined. Only crude mortality rate reported. Numerator/Denominator bias: mortality data aggregated for 2007-9 and CMR rate calculated based on 2000 Census. |
| Ichiho 2013  Majuro | Y | Y | Y | Y | NA | NA | NA | NA | NA | NA | N | N | NA | N | Poor | As for Ichiho study for Pohnpei except crude mortality rate calculated based on 1999 Census. |
| Ichiho 2013  Palau | Y | Y | Y | Y | NA | NA | NA | NA | NA | NA | N | N | NA | N | Poor | As for Ichiho study for Pohnpei except mortality data aggregated for 2006-9 and crude mortality rate calculated based on 2005 Census. |
| Ichiho 2013 Guam | Y | Y | Y | Y | NA | NA | NA | NA | NA | NA | N | N | NA | N | Poor | As for Ichiho study for Pohnpei except mortality data aggregated for 2003-5 . |
| Ichiho 2013 Northern Mariana Islands | Y | Y | Y | Y | NA | NA | NA | NA | NA | NA | N | N | NA | N | Poor | As for Ichiho study for Pohnpei except mortality data aggregated for 2003-5 |
| Ichiho 2013 American Samoa | Y | Y | Y | Y | NA | NA | NA | NA | NA | NA | N | N | NA | N | Poor | As for Ichiho study for Pohnpei except mortality data aggregated for 2006-8 |
| Ichiho 2013 Kwajelein | Y | Y | Y | Y | NA | NA | NA | NA | NA | NA | N | N | NA | N | Poor | As for Ichiho study for Pohnpei except crude mortality rate calculated based on 1990 Census. |
| Ichiho 2013 Chuuk | Y | Y | Y | Y | NA | NA | NA | NA | NA | NA | N | N | NA | N | Poor | As for Ichiho study for Pohnpei |
| Ichiho 2013  Yap | Y | Y | Y | Y | NA | NA | NA | NA | NA | NA | N | N | NA | N | Poor | As for Ichiho study for Pohnpei except mortality data aggregated for 2008-10. Diabetes reported as a separate cause of death, possible misclassification of CVD mortality as much of the mortality associated with diabetes occurs through CVD. |
| Li 1994 | Y | Y | Y | NA | Y | NA | NA | NA | NA | NA | Y | N | NA | Y | Fair | Age-adjusted prevalence provided. Outcomes measures clearly defined but unclear how subjects selected were recruited across the different sites. |
| Novak 2007 | Y | Y | Y | NA | NA | NA | NA | NA | Y | NA | NR | Y | Y | Y | Fair | Outcome measures unclear for mortality (but are defined/provided in Appendix X pg 142 for Potentially Avoidable Hospital). Study population clearly defined and specified. Data age standardised to the NZ population (Census 2001 data), which creates a numerator-denominator bias as the relevant data is 2003-5 combined data. |
| Queensland Health 2010 | Y | Y | Y | Y | NA | NA | NA | NA | N | NA | Y | Y | Y | Y | Fair | Method of determining country of birth unclear. Unclear whether ICD codes used / specified. There are standardised ratios reported. |
| Queensland Health 2011 | Y | Y | Y | Y | NA | NA | NA | NA | N | NA | Y | Y | Y | Y | Fair | Method of determining country of birth unclear. Unclear whether ICD codes specified / used. There are standardised ratios reported. |
| Reed 1970 | Y | Y | Y | N | N | NA | NA | NA | NA | NA | Y | NR | NA | CD | Poor | California values age adjusted to Guam sample but no age adjustment for Rota sample. Different recruitment methods across the three sites. |
| Taylor 1987 | Y | Y | Y | N | N | NA | NA | NA | NA | NA | Y | N | NA | Y | Poor | The populations the subjects were selected from were not comparable. Noumea sample taken from nickel factory workers (or those living nearby), whereas the Wallis sample taken from villages. Noumea population likely has healthy worker effect therefore differences are likely underestimated. No sample size justification provided, problematic as small numbers/low prevalence of different outcomes reported. |

Key: Y: Yes, N: No, NA: Not applicable, NR: Not recorded, CD: Cannot determine

## Table 3: CVD mortality (composite or by type) by Pacific-specific ethnicity or country/region of birth

| *Citation* | *CVD type* | *By country* | *By region* | *By ethnicity* | *Age-standardized mortality rate, per 100,000, unless otherwise specified* |
| --- | --- | --- | --- | --- | --- |
| *Records included in systematic review* | | | | | |
| Blakely 2009 | Composite | - | - | Samoan, Cook Islands Māori, Tongan, Niuean | CVD mortality rate, per 100,000, sexes combined (95% CI)  Cook Islands Māori: 111 (90, 135), Niuean: 75 (51-104), Samoan: 68 (57-79), Tongan: 58 (40-78)  Rate ratio compared to Samoan (95% credibility intervals)  Samoan: 1, Cook Islands Māori: 1.66 (1.26-2.13), Tongan: 0.86 (0.58-1.20), Niuean: 1.11 (0.72-1.58) |
| GBD 2019 | Atrial fibrillation and flutter | American Samoa, Cook Islands, Guam, Kiribati, Marshall Islands, Federated States of Micronesia, Nauru, Niue, Northern Mariana Islands, Palau, Papua New Guinea, Samoa, Solomon Islands, Tokelau, Tonga, Tuvalu, Vanuatu | - | - | Federated States of Micronesia: 7.08 (5.45, 9.35), Northern Mariana Islands: 6.73 (5.63, 7.83), Nauru: 6.25 (4.90, 7.96), Marshall Islands: 6.15 (4.61, 7.99), Tuvalu: 5.33 (4.22, 6.75), Niue: 5.05 (3.75, 6.36), Samoa: 4.94 (3.87, 6.22), American Samoa: 4.92 (3.92, 6.14), Tokelau: 4.87 (3.86, 6.00), Cook Islands: 4.79 (3.85, 5.82), Solomon Islands: 4.59 (3.47, 6.03), Vanuatu: 4.32 (3.20, 5.74), Kiribati: 4.29 (3.36, 5.47), Tonga: 4.14 (3.09, 5.28), Papua New Guinea: 4.01 (2.66, 5.84), Palau: 3.20 (2.53, 3.87), Guam: 3.10 (2.52, 3.72) |
|  | Hypertensive heart disease |  |  |  | Cook Islands: 65.27 (54.30, 78.06), Federated States of Micronesia: 43.23 (25.04, 58.48), Marshall Islands: 40.60 (24.27, 55.77), Solomon Islands: 34.65 (20.06, 49.21), Nauru: 33.91 (19.34, 45.22), Vanuatu: 31.98 (18.41, 43.76), Tuvalu: 30.25 (18.57, 41.75), Papua New Guinea: 29.22 (17.63, 41.36), Samoa: 27.08 (17.19, 35.22), Tokelau: 22.90 (14.63, 30.52), Niue: 21.68 (13.12, 28.79), Kiribati: 20.24 (15.47, 25.48), Guam: 16.33 (13.10, 20.47), American Samoa: 13.09 (10.73, 15.65), Tonga: 7.62 (5.69, 9.95), Northern Mariana Islands: 6.84 (5.41, 8.21), Palau: 3.16 (2.40, 3.97) |
|  | Intracerebral haemorrhage |  |  |  | Solomon Islands: 214.62 (177.64, 255.29), Kiribati: 152.17 (123.24, 183.47), Nauru: 122.68 (97.65, 152.13), Vanuatu: 120.52 (90.46, 159.00), Federated States of Micronesia: 119.53 (87.82, 152.23), Marshall Islands: 116.44 (87.33, 150.22), Papua New Guinea: 95.05 (66.44, 132.56), Tuvalu: 94.05 (74.84, 118.58), Samoa: 82.73 (67.55, 100.48), Tokelau: 66.67 (53.11, 82.56), Niue: 65.29 (51.50, 79.35), Palau: 59.42 (48.18, 73.60), American Samoa: 59.07 (51.15, 68.11), Northern Mariana Islands: 59.00 (50.84, 67.50), Tonga: 40.53 (33.30, 49.18), Cook Islands: 33.62 (27.94, 40.65), Guam: 25.32 (21.24, 30.10) |
|  | Ischemic heart disease |  |  |  | Solomon Islands: 430.35 (358.68, 501.14), Nauru: 356.23 (294.34, 424.75), Vanuatu: 304.91 (243.14, 388.28), Federated States of Micronesia: 287.23 (216.68, 365.42), Marshall Islands: 277.78 (214.38, 356.28), Tuvalu: 251.53 (201.80, 317.74), Kiribati: 249.80 (202.36, 300.91), Palau: 222.94 (180.38, 270.27), Samoa: 215.98 (181.67, 258.97), Niue: 208.81 (167.88, 247.91), Tokelau: 188.65 (154.74, 232.17), Papua New Guinea: 171.98 (126.71, 231.30), Guam: 171.76 (146.81, 200.85), American Samoa: 151.20 (131.24, 173.21), Northern Mariana Islands: 148.79 (128.85, 170.16), Tonga: 130.16 (106.02, 156.84), Cook Islands: 103.41 (87.32, 122.36) |
|  | Ischemic stroke |  |  |  | Nauru: 75.90 (60.51, 93.19), Solomon Islands: 73.92 (59.73, 91.15), Kiribati: 70.12 (56.04, 83.69), Vanuatu: 60.50 (46.07, 85.65), Federated States of Micronesia 53.66 (41.69, 66.58), Marshall Islands: 51.85 (40.00, 67.63), Palau: 51.39 (41.71, 61.97), Tuvalu: 50.51 (40.70, 63.64), Samoa: 43.39 (35.05, 52.92), Niue: 42.67 (32.55, 53.82), Tokelau: 40.31 (32.83, 49.05), American Samoa: 34.21 (28.75, 39.79), Northern Mariana Islands: 31.81 (27.05, 36.86), Tonga: 30.18 (24.27, 36.60), Papua New Guinea: 28.99 (19.46, 42.93), Cook Islands: 25.45 (20.90, 30.35), Guam: 21.51 (17.99, 25.50) |
|  | Peripheral vascular disease |  |  |  | Northern Mariana Islands: 0.60 (0.40, 0.77), Cook Islands: 0.42 (0.30, 0.55), Federated States of Micronesia: 0.39 (0.28, 0.54), Nauru: 0.37 (0.28, 0.50), Niue: 0.37 (0.28, 0.48), American Samoa: 0.34 (0.28, 0.43), Marshall Islands: 0.34 (0.24, 0.47), Samoa: 0.31 (0.23, 0.41), Tokelau: 0.29 (0.21, 0.39), Tuvalu: 0.28 (0.20, 0.40), Tonga: 0.27 (0.19, 0.36), Guam: 0.24 (0.19, 0.31), Vanuatu: 0.23 (0.16, 0.35), Kiribati: 0.22 (0.14, 0.49), Solomon Islands: 0.21 (0.15, 0.31), Papua New Guinea: 0.17 (0.12, 0.29), Palau: 0.09 (0.06, 0.13) |
|  | Stroke |  |  |  | Solomon Islands: 308.08 (259.94, 360.42), Kiribati: 238.46 (195.22, 285.69), Nauru: 208.90 (168.96, 255.05), Vanuatu: 192.78 (147.96, 258.35), Federated States of Micronesia: 186.44 (140.27, 231.34), Marshall Islands: 181.99 (139.30, 232.16), Tuvalu: 155.35 (124.86, 194.50), Samoa: 134.72 (111.67, 162.09), Papua New Guinea: 134.68 (94.77, 182.74), Palau: 116.13 (94.28, 140.79), Tokelau: 114.48 (92.52, 139.56), Niue: 113.92 (90.65, 139.60), American Samoa: 99.10 (85.18, 113.73), Northern Mariana Islands: 97.44 (84.26, 111.23), Tonga: 75.59 (63.03, 90.48), Cook Islands: 62.21 (52.88, 74.16), Guam: 50.60 (43.00, 59.92) |
|  | Subarachnoid haemorrhage |  |  |  | Solomon Islands: 19.54 (11.53, 33.01), Kiribati: 16.17 (12.28, 21.66), Marshall Islands: 13.70 (7.50, 21.16), Federated States of Micronesia: 13.26 (7.14, 20.06), Vanuatu: 11.76 (6.74, 19.27), Tuvalu: 10.80 (6.89, 16.40), Papua New Guinea: 10.65 (5.11, 18.34), Nauru: 10.32 (7.14, 14.21), Samoa: 8.61 (6.08, 11.80), Tokelau: 7.50 (5.34, 10.31), Northern Mariana Islands: 6.63 (5.26, 8.39), Niue: 5.95 (3.97, 8.40), American Samoa: 5.82 (4.65, 7.28), Palau: 5.32 (3.93, 6.82), Tonga: 4.88 (3.20, 7.18), Guam: 3.76 (3.07, 4.83), Cook Islands: 3.15 (2.08, 4.63) |
|  | Diabetes** |  |  |  | Kiribati: 203.99 (158.28, 252.68), Federated States of Micronesia: 169.13 (126.79, 226.11), Nauru: 162.80 (126.94, 227.02), Solomon Islands: 133.60 (110.61, 162.80), Niue: 122.00 (95.17, 162.49), Palau: 119.21 (92.22, 148.24), Tuvalu: 115.83 (88.37, 155.15), Marshall Islands: 112.90 (86.87, 145.94), Cook Islands: 110.42 (92.78, 130.43), Tonga: 104.61 (83.04, 129.12), Papua New Guinea: 104.02 (82.47, 130.12), American Samoa: 98.09 (84.71, 113.59), Tokelau: 96.45 (74.96, 123.15), Samoa: 89.18 (72.69, 111.28), Vanuatu: 77.53 (58.32, 103.12), Northern Mariana Islands: 58.47 (50.02, 67.70), Guam: 23.70 (19.89, 28.63) |
| Mendis 2011 | Stroke  Ischemic heart disease | Cook Islands, Kiribati, Marshall Islands, Federated States of Micronesia, Nauru, Niue, Palau, Papua New Guinea, Samoa, Solomon Islands, Tonga, Tuvalu, Vanuatu | - | - | Age standardised death rates, 2008*  Ischemic heart disease  Marshall Islands: 237.7, Papua New Guinea: 186.0, Nauru: 184.1, Tuvalu: 163.2, Samoa: 113.1, Vanuatu: 111.4, Federated States of Micronesia: 110.3, Tonga: 101.7, Palau: 101.0, Niue: 100.6, Solomon Islands: 97.9, Cook Islands: 79.1, Kiribati: 11.8  Stroke  Marshall Islands: 240.4, Tuvalu: 175.7, Kiribati: 141.5, Samoa: 125.2, Federated States of Micronesia: 121.9, Tonga: 118.6, Vanuatu: 116.1, Solomon Islands: 105.5, Papua New Guinea: 98.9, Palau: 94.8, Niue: 89.5, Nauru: 80.7, Cook Islands: 71.1 |
| WHO 2014 | Composite | Papua New Guinea, Solomon Islands | - | - | Age standardised death rate*  Males: Papua New Guinea: 179.4, Solomon Islands: 314.6  Females: Papua New Guinea: 125.3, Solomon Islands: 196.9 |
| *Records in which quality was assessed as poor or fair quality* | | | | | |
| Crews 1982 | Composite | - | Traditional: Isolated north shore of Tutuila and the Manu’a Islands.  Intermediate: Tutuila village connected to Pago Pago by paved roads.  Modern: Pago Pago harbour area | American Samoan | CVD death rate*  Total: Traditional: 316.6, Intermediate: 221.4, Modern: 294.4  Males: Traditional: 234.5, Intermediate: 275.0, Modern: 343.5  Females: Traditional: 398.7, Intermediate: 163.0, Modern: 240.9 |
| Ichiho 2013 | Diabetes**  Heart disease  Cerebrovascular accident | Federated States of Micronesia | USAPI: Yap State | - | Crude mortality rate*  Diabetes: 67.4; Heart disease: 55.7; Cerebrovascular accident: 55.7 |
| Ichiho 2013 | Myocardial infarction  Cerebrovascular accident  Hypertension** | Federated States of Micronesia | USAPI: Chuuk State | - | Crude mortality rate*  Myocardial infarction: 37.9; Cerebrovascular accident: 20.5; Hypertension: 10.6 |
| Ichiho 2013 | Myocardial infarction  Cerebrovascular accident  Heart disease  Diabetes-related** | Marshall Islands | USAPI: Kwajelein Atoll | Marshallese | Crude mortality rate*  Myocardial infarction: 39.7; Cerebrovascular accident: 15.3; Heart disease: 9.2; Diabetes-related: 82.5 |
| Ichiho 2013 | Heart disease  Cerebrovascular accident Hypertension** | American Samoa | USAPI | - | Crude mortality rate*  Heart disease: 160.6; Diabetes: 172.8; Cerebrovascular accident: 141.4; Hypertension: 48.9 |
| Ichiho 2013 | Heart disease  Stroke | Northern Mariana Islands | USAPI | - | Crude mortality rate*  Heart disease: 39.5; Stroke: 27.4 |
| Ichiho 2013 | Heart disease  Cerebrovascular accident | Guam | USAPI | - | Crude mortality rate*  Heart disease: 141.9; Cerebrovascular accident: 33.9 |
| Ichiho 2013 | Heart disease  Cerebrovascular accident | Palau | USAPI | - | Crude mortality rate*  Heart disease: 154.5; Cerebrovascular accident: 74.1 |
| Ichiho 2013 | Diabetes-related**  Hypertension**  Cardiac Arrest  Myocardial Infarction  Cerebrovascular accident | Marshall Islands | USAPI: Majuro | - | Crude mortality rate*  Diabetes-related: 42.0; Hypertension: 18.4; Cardiac arrest: 13.8; Myocardial infarction: 12.5; Cerebrovascular accident: 6.6 |
| Ichiho 2013 | Heart disease  Diabetes**  Hypertension** | Federated States of Micronesia | USAPI: Pohnpei | - | Crude mortality rate*  Heart disease: 345.1; Diabetes: 116.0; Hypertension: 66.7 |
| Novak 2007 | Ischemic heart disease  Stroke | - | - | Samoan, Cook Islands Māori, Tongan, Niuean | Age-standardised potentially avoidable mortality rate (per 100,000) in NZ Pacific people, prioritised, 15+ year olds, (2001-2003)*  Ischemic heart disease  Samoan: 232, Cook Islands Māori: 299, Tongan: 251, Niuean: 230  Stroke  Samoan: 119, Cook Islands Māori: 89, Tongan: 73, Niuean: 90 |

*No confidence interval provided or p value to indicate statistically significant difference
**Included as potentially misclassified atherosclerotic CVD related death

## Table 4: CVD hospitalisations by Pacific-specific ethnicity or country/region of birth

| *Citation* | *CVD type* | *By country* | *By ethnicity* | *Hospitalisation rate (specified)* | *Ratio or Confidence Interval* | *Additional notes* |
| --- | --- | --- | --- | --- | --- | --- |
| *Records in which quality was assessed as poor or fair quality* | | | | | | |
| Novak 2007 | Myocardial infarction  Heart failure  Stroke | - | Samoan, Cook Islands Māori, Tongan, Niuean | Age-standardised Potentially avoidable hospital rate (per 100,000)  Myocardial infarction: Samoan: 450, Cook Islands Māori: 340, Tongan: 480, Niuean: 300  Heart failure: Samoan: 420, Cook Islands Māori: 520, Tongan: 350, Niuean: 90  Stroke: Samoan: 450, Cook Islands Māori: 250, Tongan: 340, Niuean: 130 | Females  Stroke: Samoan vs Niuean ratio: 1.83, Tongan vs Niuean ratio: 1.68  Heart failure: Samoan vs Niuean ratio: 1.64, Cook Islands Māori vs Niuean ratio: 1.17  Males  Myocardial infarction: Tongan vs Cook Islands Māori ratio: 2.26  Heart failure: Samoan vs Niuean ratio: 1.43 | These data come from the top ten causes of potentially avoidable hospital rate (age-standardized) in Counties Manukau, Auckland, New Zealand for Pacific adults (15+ year olds), males and females combined (2003-5), prioritised.  Rates have large confidence intervals and are rounded to the nearest 10. Combined 2003-5 data.  ICD codes used for each cause.  *Ratio is greatest difference between relevant Pacific ethnic groups. Subject to bias due to differing widths of CIs |
| Queensland Health 2010 | Heart disease  Stroke | Samoan-born Queenslanders | Samoan | - | Standardised separation ratio: total Queensland (100)  Standardised separation ratio for Samoan-born:  Heart disease: 121.5, Stroke: 150.8 | Standardised ratio reported only.  Hospitalization rates not given.  The heart disease result should be noted in the context of the significantly higher ratios for diabetes (3x higher) in Samoan-born QLDers vs total QLDers. As diabetes increases the risk for CHD, this similar ratio for CHD should not be discounted due to statistical insignificance. |
| Queensland Health 2011 | Heart disease  Stroke | Papua New Guinea-born Queenslanders | Papua New Guinean | - | Standardised separation ratio: total Queensland (100)  Standardised separation ratio for Papua New Guinea-born:  Heart disease: 93.1  Stroke: 101.5 | Standardised ratio reported only.  Hospitalization rates not given. |

## Table 5: CVD incidence/prevalence (composite) by Pacific-specific ethnicity or country/region of birth

| *Citation* | *CVD type* | *By country* | *By ethnicity* | *Proportion of population at high risk for CVD or with existing CVD (%)* | *Additional notes* |
| --- | --- | --- | --- | --- | --- |
| WHO 2018 | Composite | Kiribati, Nauru, Solomon Islands, Tuvalu | - | Kiribati: 11, Nauru: 25, Solomon Islands: 9, Tuvalu: 13 | The total population estimates for the year 2016 were taken from the most recent United Nations Population Division World Population Prospects  These are the only Member State Pacific Islands that have data available. |

## Table 6: CVD incidence/prevalence (by type) by Pacific-specific ethnicity or country/region of birth

| *Citation* | *CVD type* | *Comparison by country/region* | *Comparison by ethnicity* | *Prevalence, age-standardized, rate per 100,000* | *Incidence, age-standardized, rate per 100,000* |
| --- | --- | --- | --- | --- | --- |
| *Records included in systematic review* | | | | | |
| GBD 2019 | Atrial Fibrillation and flutter | American Samoa, Cook Islands, Guam, Kiribati, Marshall Islands, Federated States of Micronesia, Nauru, Niue, Northern Mariana Islands, Palau, Papua New Guinea, Samoa, Solomon Islands, Tokelau, Tonga, Tuvalu, Vanuatu | - | American Samoa: 806.20 (608.34, 1040.39), Vanuatu: 799.63 (600.85, 1028.88), Tonga: 785.04 (591.14, 1008.00), Guam: 782.39 (591.68, 1008.65), Samoa: 777.35 (588.11, 999.41), Cook Islands: 777.33 (591.88, 1010.97), Palau: 770.33 (587.94, 987.11), Niue: 766.36 (580.85, 984.66), Tokelau: 763.47 (579.53, 985.76), Northern Mariana Islands: 763.11 (577.41, 975.31), Nauru: 758.19 (577.61, 979.72), Tuvalu: 752.94 (568.10, 968.82), Kiribati: 737.67 (555.72, 957.97), Marshall Islands: 726.52 (550.00, 934.08), Solomon Islands: 716.78 (541.84, 918.06), Papua New Guinea: 713.60 (536.68, 914.78), Federated States of Micronesia: 703.06 (531.31, 906.31) | Vanuatu: 64.46 (48.86, 82.64), American Samoa: 64.33 (48.79, 82.78), Tonga: 62.94 (47.20, 80.39), Samoa: 62.27 (46.94, 79.98), Guam: 62.26 (47.00, 79.85), Cook Islands: 61.55 (46.52, 79.23), Palau: 61.29 (46.51, 78.41), Niue: 61.05 (46.41, 78.30), Tokelau: 60.94 (46.48, 78.56), Northern Mariana Islands: 60.57 (45.86, 77.16), Tuvalu: 60.55 (45.76, 77.78), Nauru: 60.49 (45.66, 78.40), Kiribati: 60.42 (45.65, 78.28), Marshall Islands: 58.74 (44.47, 75.36), Papua New Guinea: 58.06 (43.98, 73.68), Solomon Islands: 57.90 (43.67, 73.72), Federated States of Micronesia: 57.05 (42.95, 73.12) |
|  | Hypertensive heart disease |  |  | Cook Islands: 703.08 (532.87, 920.72), Guam: 443.92 (320.45, 622.68), Samoa: 398.84 (287.53, 554.52), Tokelau: 398.50 (291.49, 548.70), Niue: 382.41 (276.48, 536.39), Tuvalu: 365.26 (263.01, 508.29), Federated States of Micronesia: 363.30 (261.29, 497.94), Marshall Islands: 357.84 (256.71, 502.17), Papua New Guinea: 339.72 (243.37, 475.72), Vanuatu: 336.91 (242.74, 472.95), Nauru: 328.89 (235.96, 464.47), American Samoa: 274.03 (198.18, 376.65), Solomon Islands: 267.55 (188.94, 379.88), Tonga: 254.98 (181.90, 355.74), Kiribati: 238.64 (168.97, 336.93), Northern Mariana Islands: 208.61 (148.18, 292.65), Palau: 79.37 (57.00, 110.93) | Not reported |
|  | Intracerebral haemorrhage |  |  | Kiribati: 971.74 (869.97, 1067.69), Solomon Islands: 819.25 (741.70, 900.80), Vanuatu: 818.97 (740.55, 896.89), Marshall Islands: 804.72 (731.93, 880.31), Nauru: 744.29 (677.42, 813.55), Papua New Guinea: 725.59 (656.72, 798.84), Federated States of Micronesia: 713.57 (646.87, 781.58), Samoa: 635.09 (572.03, 703.37), American Samoa: 580.06 (521.33, 636.67), Tuvalu: 557.36 (502.35, 613.18), Northern Mariana Islands: 554.48 (500.82, 612.40), Palau: 540.99 (491.60, 594.74), Niue: 527.57 (475.66, 581.27), Tokelau: 521.32 (468.63, 576.69), Cook Islands: 521.00 (471.67, 571.59), Guam: 487.41 (438.18, 536.77), Tonga: 394.32 (355.70, 437.63) | Kiribati: 161.75 (150.73, 173.76), Solomon Islands: 156.20 (143.56, 169.42), Marshall Islands: 143.98 (133.37, 155.69), Vanuatu: 142.66 (131.86, 154.76), Federated States of Micronesia: 123.93 (114.65, 135.12), Papua New Guinea: 120.71 (110.85, 130.99), Nauru: 117.87 (108.53, 128.60), Samoa: 100.94 (92.63, 110.18), Tuvalu 92.82 (85.37, 100.85), American Samoa: 80.94 (73.65, 89.00), Northern Mariana Islands: 78.28 (71.02, 86.56), Palau: 78.28 (71.71, 85.67), Tokelau: 77.14 (70.50, 85.10), Niue: 76.91 (69.96, 85.33), Cook Islands: 66.42 (59.82, 73.49), Tonga: 55.64 (50.61, 61.56), Guam: 55.07 (49.68, 61.18) |
|  | Ischemic heart disease |  |  | Vanuatu: 2738.31 (2412.48, 3139.68), American Samoa: 2620.68 (2306.16, 3000.50), Tonga: 2612.78 (2301.49, 2990.21), Samoa: 2546.00 (2234.91, 2916.29), Cook Islands: 2543.51 (2249.83, 2916.42), Guam: 2536.88 (2241.21, 2895.88), Tokelau: 2521.40 (2202.59, 2887.47), Palau: 2488.64 (2191.18, 2856.33), Papua New Guinea: 2480.68 (2194.40, 2821.08), Niue: 2463.42 (2169.73, 2821.02), Northern Mariana Islands: 2462.14 (2170.69, 2808.55), Kiribati: 2454.07 (2165.34, 2817.52), Nauru: 2438.86 (2152.60, 2796.23), Tuvalu: 2425.41 (2140.64, 2760.04), Solomon Islands: 2359.57 (2083.20, 2718.44), Marshall Islands: 2330.15 (2056.46, 2670.08), Federated States of Micronesia: 2185.09 (1924.90, 2508.65) | Vanuatu: 225.45 (195.06, 256.61), Solomon Islands: 212.99 (184.99, 243.39), Papua New Guinea: 209.79 (184.41, 237.60), Kiribati: 209.71 (182.66, 238.70), Samoa: 202.97 (176.38, 231.31), Tonga: 202.29 (174.71, 231.48), American Samoa: 200.79 (174.32, 228.58), Tuvalu: 199.58 (172.62, 227.47), Nauru: 199.15 (172.76, 227.82), Tokelau: 198.13 (171.00, 227.14), Marshall Islands: 197.36 (169.80, 226.69), Palau: 197.32 (171.74, 226.89), Niue: 192.34 (166.26, 219.17), Cook Islands: 191.68 (165.40, 219.25), Guam: 188.82 (164.23, 215.34), Northern Mariana Islands: 188.79 (163.15, 215.77), Federated States of Micronesia: 187.54 (161.42, 215.21) |
|  | Ischemic stroke |  |  | Nauru: 1360.73 (1241.00, 1500.20), Palau: 1323.85 (1202.69, 1458.49), Vanuatu: 1295.93 (1176.34, 1432.96), Guam: 1216.13 (1119.11, 1325.94), Kiribati: 1194.76 (1088.01, 1326.06), Marshall Islands: 1158.92 (1053.63, 1276.03), American Samoa: 1129.30 (1036.24, 1235.19), Cook Islands: 1128.60 (1034.26, 1234.37), Samoa: 1128.07 (1033.85, 1239.04), Niue: 1122.66 (1025.53, 1232.83), Northern Mariana Islands: 1057.44 (966.08, 1166.25), Federated States of Micronesia: 1036.19 (946.70, 1145.06), Tokelau: 1014.68 (926.22, 1114.21), Tuvalu: 1008.69 (918.33, 1111.53), Solomon Islands: 953.78 (860.83, 1049.76), Tonga: 935.38 (851.68, 1029.01), Papua New Guinea: 860.87 (782.83, 942.65) | Nauru: 122.43 (106.73, 140.81), Vanuatu: 118.98 (103.64, 135.93), Palau: 116.83 (101.79, 135.59), Kiribati: 108.82 (95.62, 124.41), Marshall Islands: 106.69 (92.29, 124.08), Samoa: 98.49 (85.20, 113.17), Niue: 95.91 (83.11, 110.62), American Samoa: 94.66 (81.76, 109.86), Guam: 92.35 (80.55, 105.74), Federated States of Micronesia: 92.17 (80.01, 105.86), Cook Islands: 91.54 (79.23, 106.56), Tuvalu: 89.68 (77.96, 102.95), Solomon Islands: 89.34 (77.17, 103.51), Northern Mariana Islands: 87.45 (76.20, 101.01), Tokelau: 86.20 (72.26, 99.37), Tonga: 78.73 (68.22, 91.37) , Papua New Guinea: 72.44 (62.38, 84.28) |
|  | Peripheral vascular disease |  |  | Kiribati: 2009.41 (1734.25, 2306.45), Samoa: 1783.91 (1542.86, 2044.54), American Samoa: 1695.93 (1466.49, 1935.03), Tonga: 1695.20 (1460.34, 1944.24), Cook Islands: 1590.67 (1374.78, 1815.44), Niue: 1589.90 (1376.42, 1814.75), Nauru: 1569.68 (1350.05, 1788.53), Palau: 1566.96 (1353.40, 1782.93), Tuvalu: 1563.76 (1352.86, 1791.38), Tokelau: 1554.57 (1339.80, 1788.16), Vanuatu: 1547.32 (1336.97, 1764.06), Federated States of Micronesia: 1498.43 (1294.44, 1722.92), Marshall Islands: 1467.44 (1269.20, 1670.83), Solomon Islands: 1462.72 (1269.66, 1676.39), Guam: 1437.83 (1239.03, 1638.44), Northern Mariana Islands: 1410.79 (1225.38, 1601.98), Papua New Guinea: 1356.00 (1173.62, 1550.64) | Kiribati: 192.39 (166.58, 220.88), Samoa: 166.77 (144.06, 192.38), Tonga: 158.71 (136.35, 181.90), American Samoa: 152.88 (132.09, 175.97), Tuvalu: 151.30 (129.97, 172.82), Vanuatu: 151.11 (131.24, 173.46), Nauru: 148.65 (127.89, 169.95), Tokelau: 146.36 (126.51, 167.65), Niue: 146.23 (125.70, 166.85), Federated States of Micronesia: 145.53 (125.70, 166.85), Solomon Islands: 145.19 (125.98, 165.69), Palau: 143.93 (125.14, 164.72), Marshall Islands: 143.24 (124.36, 164.17), Cook Islands: 140.56 (121.45, 161.37), Papua New Guinea: 132.71 (115.26, 152.36), Northern Mariana Islands: 126.56 (109.11, 145.04), Guam: 124.09 (107.36, 142.57) |
|  | Stroke |  |  | Kiribati: 2263.12 (2123.52, 2401.71), Vanuatu: 2180.29 (2042.82, 2329.19), Nauru: 2144.97 (2021.99, 2288.79), Marshall Islands: 2052.04 (1926.21, 2197.16), Palau: 1910.45 (1784.54, 2045.78), Samoa: 1841.20 (1729.09, 1971.48), Solomon Islands: 1835.60 (1728.62, 1964.34), Federated States of Micronesia: 1833.04 (1725.89, 1955.37), Guam: 1790.42 (1684.32, 1910.29), American Samoa: 1772.25 (1673.40, 1891.99), Cook Islands: 1728.09 (1622.95, 1844.50), Niue: 1712.81 (1608.93, 1834.08), Northern Mariana Islands: 1683.44 (1577.30, 1804.93), Papua New Guinea: 1682.07 (1582.08, 1792.17), Tuvalu: 1643.80 (1546.31, 1753.40), Tokelau: 1608.79 (1511.48, 1715.81), Tonga: 1416.83 (1322.55, 1519.59) | Kiribati: 296.11 (278.46, 316.57), Vanuatu: 283.69 (265.98, 304.37), Marshall Islands: 275.94 (258.57, 297.75), Solomon Islands: 266.70 (250.08, 286.71), Nauru: 257.59 (239.24, 277.84), Federated States of Micronesia: 238.21 (222.94, 256.35), Samoa: 218.80 (202.84, 236.88), Papua New Guinea: 215.79 (201.27, 231.81), Palau: 211.09 (193.28, 230.93), Tuvalu: 201.33 (187.80, 216.99), American Samoa: 191.41 (176.33, 208.02), Niue: 188.16 (173.02, 205.68), Northern Mariana Islands: 182.44 (169.44, 197.34), Tokelau: 179.54 (165.88, 194.97), Cook Islands: 175.66 (160.61, 191.75), Guam: 165.86 (152.38, 181.65), Tonga: 151.01 (138.04, 165.29) |
|  | Subarachnoid haemorrhage |  |  | Kiribati: 194.47 (171.62, 219.55), Marshall Islands: 181.65 (159.08, 207.14), Papua New Guinea: 165.69 (145.33, 187.67), Guam: 164.61 (143.26, 187.82), Federated States of Micronesia: 164.08 (144.14, 186.18), Vanuatu: 163.52 (142.64, 185.51), Samoa: 157.22 (138.81, 178.43), Cook Islands: 152.67 (133.46, 174.24), Tuvalu: 147.04 (130.07, 167.10), Tonga: 145.26 (127.07, 166.77), Solomon Islands: 144.29 (126.16, 164.96), Northern Mariana Islands: 144.18 (128.10, 163.25), American Samoa: 140.55 (124.05, 159.01), Tokelau: 139.50 (123.21, 159.36), Nauru: 137.74 (121.33, 156.34), Niue: 135.33 (118.65, 153.67), Palau: 130.70 (113.77, 150.33) | Kiribati: 25.55 (22.61, 28.86), Marshall Islands: 25.27 (22.22, 28.92), Papua New Guinea: 22.63 (19.73, 25.70), Federated States of Micronesia: 22.11 (19.40, 25.57), Vanuatu: 22.05 (19.34, 25.47), Solomon Islands: 21.16 (18.49, 24.39), Samoa: 19.37 (16.97, 22.16), Tuvalu: 18.83 (16.42, 21.59), Guam: 18.44 (14.40, 22.84), Cook Islands: 17.70 (13.81, 21.79), Nauru: 17.29 (15.31, 19.91), Northern Mariana Islands: 16.70 (14.69, 19.11), Tonga: 16.64 (13.97, 20.74), Tokelau: 16.19 (14.36, 18.73), Palau: 15.98 (12.92, 20.14), American Samoa: 15.81 (13.85, 18.28), Niue: 15.35 (13.29, 18.48) |
| *Citation* | *CVD type* | *Comparison by country/region* | *Comparison by ethnicity* | *Incidence / prevalence CVD (by type)* | *Ratio, P-value, or confidence interval* |
| Health Partners Consulting Group 2012 | Ischemic heart disease  Stroke  Heart failure  Peripheral vascular disease | - | Niuean,  Cook Islands Māori, Tongan, Samoa | Estimated CVD (by type) prevalence by Pacific constructed population, all ages, Auckland metro DHBs 2011  Age-standardized rates per 1000 population  Ischemic heart disease  Total: Niue: 107.8, Cook Islands Māori: 138.0, Tonga: 112.8, Samoa: 118.1; Males: Niue: 112.0, Cook Islands Māori: 135.0, Tonga: 112.2, Samoa: 116.3; Females: Niue: 104.0, Cook Islands Māori: 140.3, Tonga: 113.2, Samoa: 119.6  Stroke  Total: Niue: 10.3, Cook Islands Māori: 12.9, Tonga: 9.9, Samoa: 13.5; Males: Niue: 14.0, Cook Islands Māori: 13.8, Tonga: 9.7, Samoa: 14.2; Females: Niue: 8.0, Cook Islands Māori: 12.2, Tonga: 10.0, Samoa: 13.0  Heart failure  Total: Niue: 20.3, Cook Islands Māori: 34.5, Tonga: 23.8, Samoa: 23.3; Males: Niue: 19.1, Cook Islands Māori: 37.7, Tonga: 26.5, Samoa: 25.5; Females: Niue: 20.6, Cook Islands Māori: 32.2, Tonga: 21.4, Samoa: 21.5  Peripheral vascular disease  Total: Niue: 5.3, Cook Islands Māori: 7.0, Tonga: 5.1, Samoa: 4.9; Males: Niue: 8.4, Cook Islands Māori: 9.0, Tonga: 5.6, Samoa: 5.9; Females: Niue: 3.3, Cook Islands Māori: 5.4, Tonga: 4.6, Samoa: 3.9 | Ischemic heart disease  Cook Island total rate: P<0.001  Stroke  Tonga age-standardized rates: P<0.05  Samoan age-standardized rates: P<0.05  Heart failure  Niuean age-standardized rates: P<0.05  Cook Islands Māori age-standardized rates: P<0.01  Peripheral vascular disease  Cook Islands Māori age-standardized rates: P<0.05 |
| *Records in which quality was assessed as poor or fair quality* | | | | | |
| Bennett 1984 | Heart disease | Rarotonga (Cook Islands), Niue | - | Age-adjusted prevalence (%) of electrocardiographic changes suggestive of heart disease  Males: Rarotonga: 5.4, Niue: 1.1; Females: Rarotonga: 1.1, Niue1.3 | Males: Niue prevalence P<0.05; Females: Niue prevalence: not significant for Niue < Rarotonga |
| Li 1994 | Heart disease | Cook Islands, Niue, Western Samoa, Fiji (Lakeba), New Caledonia (Loyalty), New Caledonia (T, O, N, W), Kiribati, Nauru | Polynesian (Cook Islands, Niue, Western Samoa), Melanesian / Polynesian (Fiji, New Caledonia), Micronesian (Kiribati, Nauru) | Q and ST-T together (age-adjusted)  Men  Cook Islands – Rarotonga: 6.2, Niue: 3.3, Western Samoa: 2.9, Fiji (Lakeba): 0.8, New Caledonia (Loyalty): 9.8, New Caledonia (T, O, N, W): 8.0, Melanesian – Fiji: 8.7, Kiribati: 6.7, Nauru: 7.3  Women  Cook Islands – Rarotonga: 19.3, Niue: 10.8, Western Samoa: 11.9, Fiji (Lakeba): 11.9, New Caledonia (Loyalty): 14.4, New Caledonia (T, O, N, W): 14.8, Melanesian – Fiji: 18.7, Kiribati: 26.3, Nauru: 5.5 | 95% CIs for corresponding age-adjusted Q and ST-T together prevalence  Men  Cook Islands – Rarotonga: 2.2, 10.3; Niue: 0, 7.6; Western Samoa: 0, 6.9; Fiji (Lakeba): 0, 7.2; New Caledonia (Loyalty): 0, 20.9; New Caledonia (T, O, N, W): 4.7, 11.4; Melanesian – Fiji: 4.8, 12.6; Kiribati: 4.0, 9.3; Nauru: 2.9, 11.7  Women  Cook Islands – Rarotonga: 15.3, 23.1; Niue: 6.9, 14.7; Western Samoa: 8.3, 15.5; Fiji (Lakeba): 5.3, 18.4; New Caledonia (Loyalty): 5.8, 23.0; New Caledonia (T, O, N, W): 11.1, 18.4; Melanesian – Fiji: 14.9, 22.4; Kiribati: 23.6, 28.9; Nauru: 1.3, 9.5 |
| Reed 1970 | Heart disease  Stroke  Angina  Myocardial infarction  Heart failure  Intermittent claudication  Cerebrovascular accident | Rota, Guam, California | Chamorros | Percent positive response on symptom questionnaire by sex and area  Doctors diagnosis of heart disease  Male: California: 6, Guam: 3, Rota: 2, Female: California: 9, Guam: 6, Rota: 1  Doctors diagnosis of stroke  Male: California: 1, Guam: 2, Rota: 0, Female: California: 1, Guam: 1, Rota: 1  Percent of subjects with an interview history of illness, by sex and area  Angina pectoris  Male: California: 2, Guam: 3, Rota: 3, Female: California: 3, Guam: 2, Rota: 1  Myocardial Infarction  Male: California: 4, Guam: 2, Rota: 0, Female: California: 0, Guam: 1, Rota: 0  Heart Failure  Male: California: 5, Guam: 1, Rota: 1, Female: California: 8, Guam: 3, Rota: 0  Intermittent Claudication  Male: California: 2, Guam: 1, Rota: 0, Female: California: 1, Guam: 0, Rota: 0  Cerebrovascular accident  Male: California: 1, Guam: 2, Rota: 0, Female: California: 2, Guam: 1, Rota: 1  Percent of subjects by disease variables, area and sex  Class I ECG abnormality (suggestive of ischemic heart disease)  Male: California: 6, Guam: 2, Rota: 1, Female: California: 4, Guam: 1, Rota: 0 | - |
| Taylor 1987 | Ischemic heart disease | Wallis Island, Noumea | Wallisians | Age-standardized prevalence (%) of ECG changes in Wallis Polynesians aged 25-64 years  Q waves  Males: Wallis: 1.9, Noumea: 3.8, Females: Wallis: 0.6, Noumea: 4.3 | P<0.05 for Noumea > Wallis females  The difference in rates between males in Noumea and Wallis was not statistically significant. |

## Table 7: Gaps in use (prescription, dispensing or adherence) of triple therapy treatment (antiplatelet, antihypertensive and lipid lowering therapy) among people with CVD by Pacific-specific ethnicity or country/region of birth.

| *Citation* | *CVD type* | *By country* | *By ethnicity* | *Proportion of high risk persons receiving any drug therapy and counselling to prevent heart attacks and strokes (%)* | *Additional notes* |
| --- | --- | --- | --- | --- | --- |
| *Records included in systematic review* | | | | | |
| WHO 2018 | Heart attacks and stroke combined | Kiribati, Nauru, Solomon Islands, Tuvalu | - | Kiribati: 29, Nauru: 30, Solomon Islands: 11, Tuvalu: 43 | The denominator is the population at high risk for CVD or with CVD (reported in an earlier outcome). |

## Table 8: Gaps in use of CVD investigations and interventions delivered in secondary care (specifically angiography, percutaneous stenting, CABG) by Pacific-specific ethnicity or country/region of birth.

| *Citation* | *CVD type* | *By country* | *By ethnicity* | *Angiography* | *Angioplasty* | *Percutaneous stenting* | *Coronary artery bypass grafts* | *Additional notes* |
| --- | --- | --- | --- | --- | --- | --- | --- | --- |
| *Records in which quality was assessed as poor or fair quality* | | | | | | | | |
| Novak 2007 | - | In New Zealand | Samoan, Cook Islands Māori, Tongan, Niuean | Assessment rate was between 300-400 (per 100,000) for each group | Surgical rate was between 60-100 (per 100,000) for each group | - | Surgical rate was between 40-70 (per 100,000) for each group | All figures are approximations (read off graph)  No statistically significant differences shown in either gender between any of the Pacific ethnic groups for the intervention rates for angiography, angioplasty, or CABG. |
